# Dermal IRF4+ dendritic cells and monocytes license CD4+ T helper cells to distinct cytokine profiles

**DOI:** 10.1038/s41467-020-19463-9

**Published:** 2020-11-06

**Authors:** Kerry L. Hilligan, Shiau-Choot Tang, Evelyn J. Hyde, Elsa Roussel, Johannes U. Mayer, Jianping Yang, Kirsty A. Wakelin, Alfonso J. Schmidt, Lisa M. Connor, Alan Sher, Andrew S. MacDonald, Franca Ronchese

**Affiliations:** 1 Malaghan Institute of Medical Research, Wellington, 6012 New Zealand; 2 Department of Pathology and Molecular Medicine, University of Otago Wellington, Wellington, 6021 New Zealand; 3 Immunobiology Section, Laboratory of Parasitic Diseases, National Institute of Allergy and Infectious Diseases, National Institutes of Health, Bethesda, 20892 MD USA; 4 Lydia Becker Institute of Immunology and Inflammation, Manchester Collaborative Centre for Inflammation Research, Faculty of Biology, Medicine and Health, University of Manchester, Manchester Academic Health Science Centre, Manchester, UK; 5 Present Address: School of Biological Sciences, Victoria University of Wellington, Wellington, 6012 New Zealand

**Keywords:** Antigen-presenting cells, Antimicrobial responses, Dendritic cells, CD4-positive T cells

## Abstract

Antigen (Ag)-presenting cells (APC) instruct CD4+ helper T (Th) cell responses, but it is unclear whether different APC subsets contribute uniquely in determining Th differentiation in pathogen-specific settings. Here, we use skin-relevant, fluorescently-labeled bacterial, helminth or fungal pathogens to track and characterize the APC populations that drive Th responses in vivo. All pathogens are taken up by a population of IRF4+ dermal migratory dendritic cells (migDC2) that similarly upregulate surface co-stimulatory molecules but express pathogen-specific cytokine and chemokine transcripts. Depletion of migDC2 reduces the amount of Ag in lymph node and the development of IFNγ, IL-4 and IL-17A responses without gain of other cytokine responses. Ag+ monocytes are an essential source of IL-12 for both innate and adaptive IFNγ production, and inhibit follicular Th cell development. Our results thus suggest that Th cell differentiation does not require specialized APC subsets, but is driven by inducible and pathogen-specific transcriptional programs in Ag+ migDC2 and monocytes.

## Abstract (web summary)

Antigen presenting cells induce CD4+ T helper (Th) differentiation upon pathogen encounters. Here the authors use fluorescently-labeled bacteria, helminth and fungi to track and describe the functions of IRF4+ migratory type 2 dendritic cells and monocytes in the specific induction of Th1, Th2 or Th17 responses following skin inoculation.

## Introduction

The differentiation of CD4+ T cells into distinct functional populations is a key event in adaptive immunity: IFNγ+ T helper (Th)1 cells clear intracellular pathogens and some bacterial infections, while IL-4+ Th2 and IL-17+ Th17 cells respond to helminth and fungal infections, respectively^1^. The context of antigen recognition is a major factor in determining CD4+ T cell differentiation^2,3^. Through the expression of pattern recognition and cytokine receptors, antigen-presenting cells (APCs) perceive pathogens while sensing the response of bystander cells to the same stimuli^4^, thereby conveying the sum of these signals to initiate the appropriate CD4+ T cell differentiation program.

Dendritic cells (DC) are considered the primary APC population capable of priming CD4+ T cell responses in vivo^5^. They comprise two main populations: IRF8+ DC1 and IRF4+ DC2, which can be either lymphoid tissue-resident or migratory^6,7^. In addition, epidermal Langerhans cells (LC) and blood-borne classical Ly6C^hi^ monocytes may also participate in driving CD4+ T cell responses. The presence of such a diverse repertoire of APC has led to the proposal that APC populations may specialize in promoting certain CD4+ T cell differentiation programs, a notion that is supported by the differential expression of key cytokines and co-stimulatory molecules by different DC subsets (reviewed in ref. ^8^). Mouse models lacking specific populations of APC also suggest a similar conclusion, with DC1 driving IFNγ+ CD4+ T cell responses and KLF4-dependent DC2, NOTCH2-dependent DC2 and LC implicated in promoting the differentiation of IL-4+ or IL-17+, but not IFNγ+ CD4+ T cells^8–11^. Depending on the model and environment, monocytes favor the differentiation of IFNγ+ CD4+ T cells, but can also support allergic inflammation or anti-fungal Th17 responses (reviewed in ref. ^12^).

Besides individual APC populations having a role in driving specific types of CD4+ T cell responses, there is also evidence that APC exposed to appropriate stimuli can display a degree of functional plasticity to induce different types of responses^13–16^. This evidence stems mainly from in vitro culture experiments, although some in vivo evidence also exists^17^. Stimuli are thought to act mostly via induction of specific polarizing cytokines^1^, but also by tuning the strength of TCR-signaling via modulation of TCR ligands and costimulatory molecule expression on APC^18^.

While there is good experimental evidence supporting both DC specialization and plasticity, few studies have addressed this question in a systematic fashion. To date, most studies have employed either in vitro culture models, or in vivo models focused on individual pathogens in specific tissues, with overarching conclusions drawn from the collation of several in vivo studies performed using different readouts, tissues, and models. When different models of Th response were compared in vivo, they were often based on protein antigen combined with adjuvants such as polyI:C, CpG or alum, which are unlikely to recapitulate the localization, distribution, and innate activating properties of complex particulate pathogens such as bacteria, fungi, or multicellular parasites.

In this study, we formally test the notion of APC plasticity by comparing the role of APC subsets in mice immunized via the same intradermal route with different skin-relevant pathogens. By using mutant mouse strains that are defective in either migDC2 or monocytes, we report that the same population of dermal migDC2 can take up different pathogens and support the optimal differentiation of CD4+ T cells producing IFNγ, IL-4, or IL-17A, whilst monocytes are essential for early innate IFNγ production and full Th1 differentiation after bacterial immunization. Therefore, pathogen signals activate dermal migDC2 to express specific transcriptional programs that support the differentiation of Th cells into diverse functional phenotypes.

## Results

### Ag+ DC2 in the lymph node of mice immunized with different pathogens express similar surface markers

To investigate the APC requirements for Th1, Th2, and Th17 development, we used the skin-relevant pathogenic or opportunistic microorganisms *Mycobacterium smegmatis* (*Ms*)*, Nippostrongylus brasiliensis* (*Nb*) and *Candida albicans* (*Ca*), which induce CD4 + T cell responses that are characterized by production of IFNγ, IL-4, and IL-17A, and expression of the transcription factors Tbet, GATA-3 and RORγt, respectively (Supplementary Fig. 1a, b)^19,20^. Although IL-17A expression was observed only after *Ca* immunization, other cytokines especially IFNγ are also expressed in this model^19^.

**Fig. 1:**
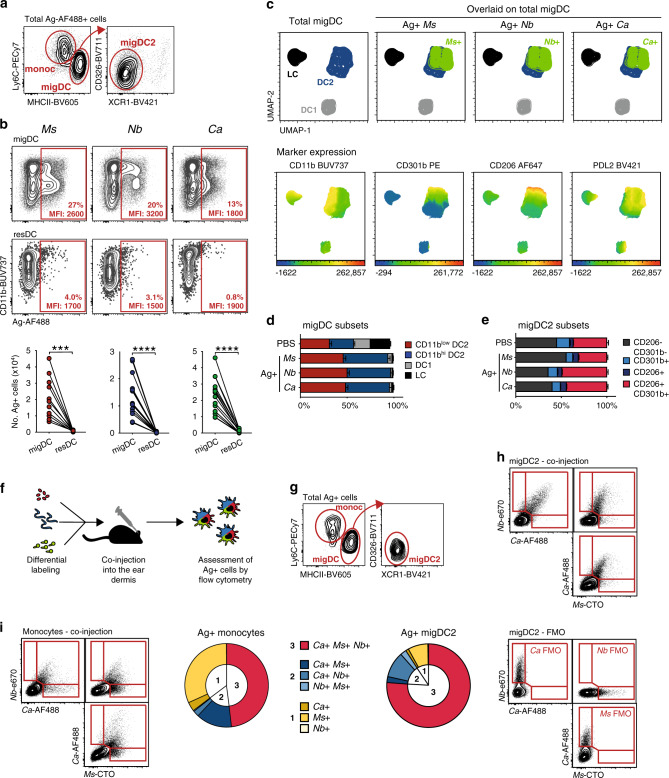
Different types of pathogens can be simultaneously taken up by the same population of dermal migDC2. C57BL/6 mice were immunized i.d. with fluorescently labeled *Ms*, *Nb*, *Ca*, or PBS as a control. Flow cytometry analysis was performed on LN cell suspensions 2 days after immunization. **a** Cellular composition and gating of the Ag+ population across treatment groups. **b** Uptake of different pathogens by migDC and resDC. Each symbol corresponds to one mouse. Data refer to groups of *n* = 11 (*Ms*) or 15 (*Nb*, *Ca*) female mice examined over 3 (*Ms*) or 4 (*Nb*, *Ca*) independent experiments. Statistical significance was assessed by paired two-tailed Student’s *t* test. ***p < 0.001, ****p < 0.0001. **c** UMAP analysis of concatenated multi-color flow cytometry data pre-gated on migDC from 4 immunized female mice per group. Analysis was performed on 161,125 cells using 11 parameters (FSC-A, SSC-A, CD11c, CD11b, CD103, CD206, CD301b, CD326, PDL2, MHCII, Ag-AF488) with the FlowJo 10 default settings (nearest neighbors = 15; minimum distance = 0.5; Euclidean distance function). Top panels highlight Ag+ cells (in green) from each treatment group overlaid on total migDC. Lower panels highlight the expression of CD11b, CD301b, CD206, and PDL2 across migDC populations using a rainbow heatmap scale. Data are representative of two independent experiments that gave similar results. **d**, **e** Distribution of Ag+ cells in migDC (**d**) and migDC2 (**e**) subsets across treatment groups compared to PBS-injected mice. Bar graphs in (**d**) show mean ± SEM for *n* = 17 (PBS), 20 (*Ms*), or 19 (*Nb*, *Ca*) female mice over five independent experiments. Bar graphs in (**e**) show mean ± SEM for groups of *n* = 9 (*Ms* and *Nb*) or 10 (PBS and *Ca*) female mice over two independent experiments. **f** Experimental outline and (**g**) gating strategy for assessing antigen uptake in mice co-injected with *Ms*-CTO, *Nb*-e670, and *Ca*-AF488. **h**, **i** Flow plots showing the relationship between *Ms*-CTO+, *Nb*-e670+, and *Ca*-AF488 + migDC2 (**h**) and monocytes (**i**). Pie charts summarize the proportion of cells positive for 1, 2, or 3 pathogens among total Ag+ migDC2 and monocytes. Data refer to groups of *n* = 5 female mice from one of 3 independent experiments that gave similar results. Source data are provided as a Source Data file.

Intradermal (i.d.) injection of inactivated, fluorescently-labeled *Ms*, *Nb,* or *Ca* enabled the identification of Ag+ cells in the ear-draining lymph node (dLN) of recipient mice on day 1-3 (Supplementary Fig. 1c). On day 2, these cells were predominantly migDC2 (MHCII^hi^ Ly6C- XCR1− CD326−) and classical monocytes (Ly6C^hi^ CD11b+) with rare resDC (Fig. 1a–c, Supplementary Fig. 1c, d), an identity which is confirmed by single-cell RNAseq data^19^. A UMAP analysis of flow cytometry data examining the migDC markers CD11b, CD11c, CD103, CD206, CD301b, CD326, PDL2, MHCII, and Ag-AF488, showed that Ag+ migDC from all conditions fell into two major clusters: CD11b^hi^ migDC2 and CD11b^low^ migDC2 (which we previously termed triple-negative, or “TN”^21^) (Fig. 1c) that were equally represented across all conditions (Fig. 1d). Within the Ag+ CD11b^hi^ and CD11b^low^ clusters, all cells were PDL2+ and a proportion co-expressed the migDC2 markers CD301b and CD206 (Fig. 1c, e, Supplementary Fig. 1d).

To assess whether different pathogens were taken up by yet unidentified but distinct subsets of migDC2, each pathogen was labeled with a different fluorophore and co-injected i.d. (Fig. 1f). As with single pathogen injections, Ag+ cells were predominantly migDC2 and monocytes (Fig. 1g). Over 75% of Ag+ migDC2 took up all three pathogens, demonstrating that individual migDC2 had no preference for particular pathogens (Fig. 1h). Among monocytes, almost 50% of cells took up all three pathogens, indicating that, when present, monocytes are capable of taking up material from multiple sources (Fig. 1i).

Small pathogens such as *Ms* (3–5 µm) may be able to drain to LN and be taken up by LN-resident DC and/or macrophages^22^. At 6 h and 12 h after *Ms* injection, the total number of Ag+ cells in dLN was low and mostly comprised of neutrophils as well as monocytes and migDC. Similar results were obtained in *Nb*- and *Ca*-treated mice 12 h post-injection, except that *Nb*+ neutrophils were rare (Supplementary Fig. 1e, f), suggesting that direct drainage accounted only for a small proportion of Ag+ cells in LN.

Therefore, our studies could not identify DC or monocyte subsets that specialize in taking up different types of pathogens.

### Upregulation of cytokine transcripts in Ag+ migDC2 is condition-specific, whereas upregulation of co-stimulatory molecules is non-specific

**Fig. 2:**
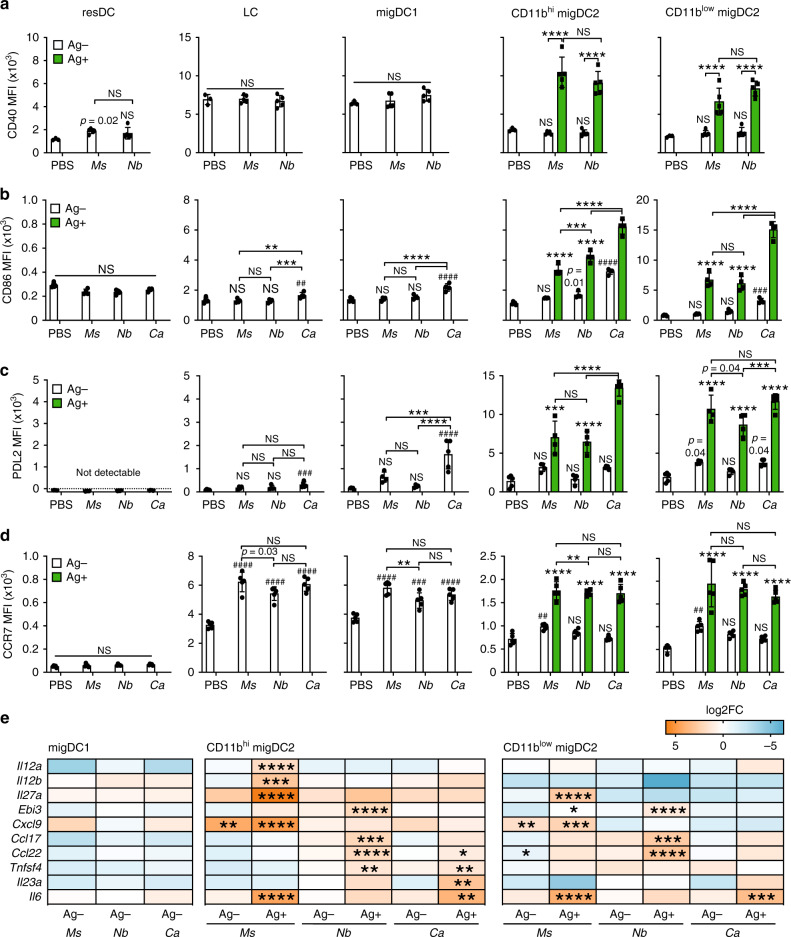
Ag+ DC express high levels of activation markers and transcripts associated with Th cell polarization. C57BL/6 mice were immunized i.d. with fluorescently labeled *Ms*, *Nb*, *Ca*, or PBS as a control. Flow cytometry (**a**–**d**) and cell sorting (**e**) were performed on LN cell suspensions 2 days later. Bar graphs show mean median fluorescence intensity (MFI) ± SD, each symbol corresponds to one mouse. **a**–**d** Expression of CD40, CD86, PDL2, and CCR7 across Ag+ and Ag− DC subsets from  PBS-injected or immunized mice. Group sizes are; *n* = 3 (CD40 PBS), 4 (CD86 immunized, PDL2 *Ms* and PDL2 *Nb*) or 5 (CD40 immunized, CD86 PBS, PDL2 PBS, PDL2 *Ca* and all CCR7) female mice from 1 of 2 (CD40, PDL2, and CCR7) or 3 (CD86) independent experiments that gave similar results. Statistical significance was assessed using Two-Way ANOVA with Sidak’s post-test. Hash symbols above individual columns refer to comparisons to the PBS group. NS: not significant; **^,##^*p* < 0.01; ***^,###^*p* < 0.001; ****^,####^*p* < 0.0001. Exact values are shown for 0.05 > *p* > 0.01. **e** Dynabead-enriched DC were sorted into Ag− and Ag+ subsets for RT-qPCR analysis. Heatmaps show differential expression analysis (log2 fold change) of transcripts in DC subsets from immunized mice compared to PBS controls. Data refer to three biological replicates each containing cells sorted from pooled LN from five female mice. Statistical significance was assessed using One-Way ANOVA with Holm–Sidak’s post-test; *p < 0.05, **p < 0.01, ***p < 0.001, ****p < 0.0001. Source data are provided as a Source Data file.

We examined DC expression of a small panel of co-stimulatory molecules including CD40 and PDL2 which have been preferentially associated with Th responses of different phenotypes^23,24^. At two days after immunization, Ag+ migDC2 had markedly upregulated CD40, CD86, and PDL2 compared to Ag− migDC2 in the same LN, or migDC2 in PBS-treated mice (Fig. 2a–c). *Ca*+ migDC2 expressed the highest levels of CD86 and PDL2. *Ms*+ and *Nb*+ migDC2 expressed similarly high levels of CD86, PDL2, and CD40, whereas other DC populations in the same LN, including resDC, LC, and migDC1 populations, expressed levels that were either comparable to PBS controls, or less elevated than in Ag+ migDC2 from the same mouse (Fig. 2a–c). Some “bystander” upregulation of CD86 and PDL2 on Ag- DC was also observed, especially in *Ca*-immunized mice, which was presumably due to secretion of inflammatory cytokines in the skin or LN^25^. Upregulation of CCR7 was most pronounced in the Ag+ compartment; some upregulation was also observed on all migDC subsets from immunized mice compared to PBS controls, but not on resDC (Fig. 2d). The comparable upregulation of CCR7 suggests similar localization of Ag+ migDC2 within the dLN (reviewed in^26^).

To assess the expression of genes that may be functionally relevant to the induction of different CD4+ Th responses, we performed RT-qPCR on Ag+ and Ag− DC subsets sorted from LN according to the strategy in Supplementary Fig. 2. In line with Fig. 2a–d, changes in gene expression were mostly restricted to the Ag+ migDC2 populations (Fig. 2e), in particular the CD11b^hi^ migDC2 subset. However, unlike the expression of co-stimulatory molecules, the transcriptional profile of Ag+ migDC2 was condition-specific (Fig. 2e): *Il12a*, *Il12b*, *Il27,* and *Cxcl9* were specifically upregulated by *Ms*+ migDC2, whereas *Ccl17*, *Ccl22,* and *Tnfsf4* (encoding OX40L) were preferentially expressed by *Nb*+ migDC2, and *Il6* and *Il23a* were upregulated by *Ca*+ migDC2.

Therefore, uptake of different types of pathogens is associated with comparable upregulation of costimulatory molecules by Ag+ DC, but differential expression of gene modules associated with the development of distinct Th effector phenotypes^19,27^.

### MigDC2 are necessary for the induction of optimal CD4+ Th responses following intradermal immunization

**Fig. 3:**
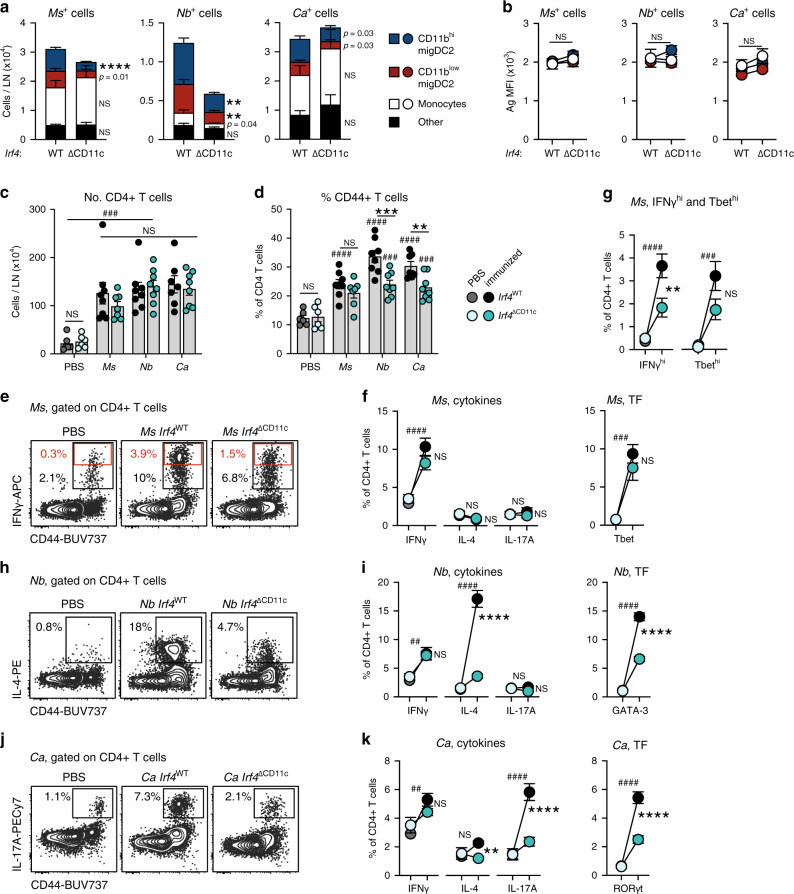
MigDC2 are required for optimal IFNγ, IL-4, and IL-17A responses by CD4+ T cells. IRF4^WT^→ or IRF4^∆CD11c^→ C57BL/6 bone marrow chimeras were immunized i.d. with *Ms*, *Nb*, *Ca*, or PBS as a control. LN cell suspensions were examined by flow cytometry on day 2 (**a**, **b**) or day 5 (**c**–**k**) after immunization. All conditions were compared within each experiment. **a**, **b** Cellular composition (**a**) and Ag median fluorescence intensity (MFI) (**b**) of LN Ag+ populations 2 days after immunization with CellTracker Orange-labeled *Ms*, *Nb,* or *Ca*. Graphs show mean ± SEM for groups of *n* = 9 (*Ms* and *Nb* IRF4^WT^) or 8 (*Ca* IRF4^WT^ and all IRF4^∆CD11c^) female chimeras from two independent experiments. Statistical significance was assessed using a two-sided Student’s *t* test for each cell population. NS: not significant, **p < 0.01, ****p < 0.0001. Exact values are shown for 0.05 > *p* > 0.01. **c**–**k** CD4+ T cell responses as assessed by flow cytometry. Intracellular cytokine staining was after in vitro restimulation, transcription factor (TF) expression was assessed without in vitro restimulation. **c** Numbers of CD4+ T cells per LN and (**d**) frequencies of CD44+ cells in the indicated CD4+ T cell populations. Bar graphs show mean values ± SEM, each symbol corresponds to one mouse. **e** Contour plots showing IFNγ staining of CD4+ T cells from *Ms*-immunized mice. Data are concatenated from 4 mice. **f** Frequencies of total cytokine+ and Tbet+ CD4+ T cells, or (**g**) IFNγ^hi^ (red gates in (**e**)) and Tbet^hi^ CD4+ T cells, in *Ms*-immunized mice. **h**, **j** Contour plots showing IL-4 and IL-17A staining of CD4+ T cells from *Nb*- or *Ca-*immunized mice, respectively. Data are concatenated from 4 mice. **i**, **k** Frequencies of cytokine+ and TF+ CD4+ T cells in *Nb-* or *Ca*-immunized mice. All graphs show mean ± SEM for groups of *n* = 6 (PBS IRF4^WT^ and PBS IRF4^∆CD11c^) *n* = 7 (*Ca* IRF4^WT^ and *Ms* IRF4^∆CD11c^) or *n* = 8 (*Ms, Nb* IRF4^WT^ and *Nb, Ca* IRF4^∆CD11c^) female chimeras over two independent experiments; each symbol in (**c**, **d**) corresponds to one mouse. Statistical significance was assessed using Two-Way ANOVA with Sidak’s post-test. NS: not significant; ^##,^**p < 0.01; ^###,^***p < 0.001; ^####,^****p < 0.0001. Hash symbols refer to comparisons between PBS and immunized chimeras of the same genotype. Asterisks refer to comparisons between similarly immunized IRF4^WT^ or IRF4^∆CD11c^ chimeras. Source data are provided as a Source Data file.

To assess whether Ag uptake by APC populations predicts their role in driving CD4+ T cell responses in LN, we first used chimeric mice reconstituted with *Itgax*-Cre+ *Irf4*^fl/fl^ (IRF4^ΔCD11c^) or *Itgax*-Cre- *Irf4*^fl/fl^ (IRF4^WT^) bone marrow. In these mice, resDC, LC, and migDC1 develop normally, whereas migDC2 numbers are reduced due to a defective ability to migrate to the dLN^23,28^ (Supplementary Fig. 3a). Due to the expression of IRF4-GFP in IRF4^ΔCD11c^ mice, pathogens were labeled with CellTracker Orange to differentiate them from the endogenous GFP signal. As expected, the numbers of *Ms*+, *Nb*+, and *Ca*+ migDC2 were lower in IRF4^ΔCD11c^ mice compared to IRF4^WT^ controls (Fig. 3a). Nonetheless, total *Ms*+ or *Ca*+, but not *Nb*+, cell numbers remained high due to the uptake of *Ms* and *Ca* by monocytes and other cell populations (Fig. 3a). Antigen uptake per cell was also similar between IRF4^ΔCD11c^ and IRF4^WT^ mice (Fig. 3b), whereas CD86 expression was lower in *Ms*+ or *Ca*+ migDC2 from IRF4^ΔCD11c^ mice compared to IRF4^WT^ (Supplementary Fig. 3b). The number and phenotype of *Ms*+ monocytes and their expression of the IFNγ-dependent markers CD11c and Ly6A/E on day 2 and 3 was also not affected by conditional IRF4 deletion (Supplementary Fig. 3c).

We then examined CD4+ T cell responses in IRF4^ΔCD11c^ and IRF4^WT^ mice 5 days after immunization. Despite reduced Ag+ DCs in dLN, numbers of CD4+ T cells and percentages of CD44^hi^ CD4+ T cells were increased in all immunized mice compared to PBS regardless of genotype (Fig. 3c, d), suggesting that a T cell response was being generated. However, there was a trend to lower percentages of CD44^hi^ CD4+ T cells in all immunized IRF4^ΔCD11c^ mice compared to IRF4^WT^, which reached statistical significance in the *Nb-* and *Ca*-immunized groups (Fig. 3d). IRF4^ΔCD11c^ and IRF4^WT^ mice immunized with *Ms* generated similar frequencies of total IFNγ+ and Tbet+ CD4+ T cells, and very low frequencies of IL-4+ and IL-17A+ CD4+ T cells (Fig. 3e, f). However, high IFNγ- and Tbet-expressing CD4+ T cells were more frequent in IRF4^WT^ mice compared to IRF4^ΔCD11c^, suggesting that migDC2 were necessary to support optimal Th1 differentiation (Fig. 3g). *Nb* and *Ca* immunization of IRF4^WT^ mice led to increased percentages of IL-4+ and IL-17A+ CD4+ T cells, respectively, with similar increases in the expression of GATA-3 and RORγt. These increases were markedly reduced in IRF4^ΔCD11c^ mice, with no increase in the expression of other cytokines (Fig. 3h–k), indicating that migDC2 were essential for Th2 and Th17 differentiation after skin immunization.

To assess the role of other migDC populations, we measured Th responses in diphtheria toxin (DT)-treated Langerin-DTR and in BATF3-KO mice that lack LC and migDC1, respectively (Supplementary Fig. 4a). Both of these populations express *Il12b* transcripts in the steady state (Immgen.org), and have been reported to be either necessary for Th1 development or suppressive for Th2 development in some models^8^. In all cases, LN cellularity, proportions of CD4+ CD44^hi^ cells, and percentages of IFNγ+, IL-4+, and IL-17A+ CD4+ T cells were at least as high in mutant mice as in controls, indicating that LC and migDC1 were not required for the CD4+ T cell cytokine response to *Ms*, *Nb,* or *Ca* (Supplementary Fig. 4b–g). Higher LN cellularity and frequency of IL-4+ CD4+ T cells were observed in BATF3-KO mice immunized with *Ms* or *Nb*, respectively. Given the overall normal Th response in Langerin-DTR and BATF3-KO mice, no in vivo antigen uptake studies were carried out in these mice.

Therefore, Ag uptake and T cell cytokine expression studies both identify migDC2 as the APC population driving the development of optimal IFNγ, IL-4, and IL-17A responses, although the requirement for migDC2 is less stringent in the case of IFNγ.

### Monocytes are necessary for the differentiation of IFNγ+ CD4+ T cells following Ms immunization

**Fig. 4:**
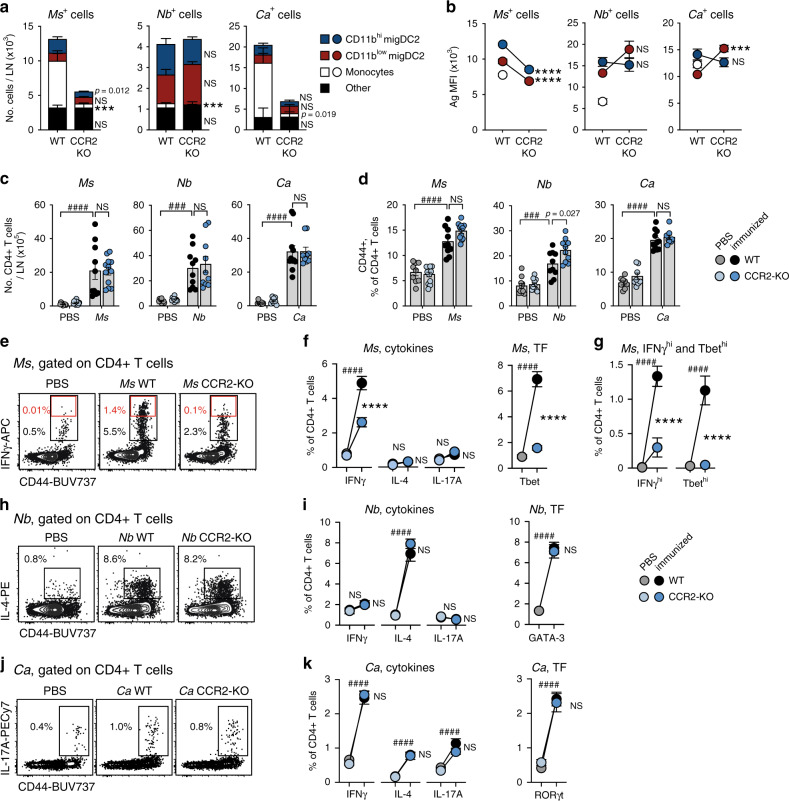
CCR2-dependent monocytes are necessary for IFNγ production by CD4+ T cells after *Ms* immunization. C57BL/6 (WT) and CCR2-KO mice were immunized i.d. with *Ms*, *Nb*, *Ca*, or PBS as a control. LN cell suspensions were examined by flow cytometry on day 2 (**a**, **b**) or 5 (**c**–**k**) as indicated. **a**, **b** Cellular composition (**a**) and Ag median fluorescence intensity (MFI) (**b**) of LN Ag+ populations after immunization with AF488-labeled *Ms*, *Nb* or *Ca*. Monocyte MFI values for CCR2-KO mice are not shown due to low monocyte counts. Graphs show mean ± SEM for groups of n = 8 (4 female + 4 male, *Ms* WT and *Ms* KO), 8 male (*Nb* WT, *Ca* WT and *Ca* KO), or 10 male (*Nb* KO) mice over two independent experiments. Statistical significance was assessed using a two-sided Student’s *t* test for each cell population. NS: not significant, ***p < 0.001, ****p < 0.0001. Exact values are shown for 0.05 > *p* > 0.01. **c**–**k** CD4+ T cell responses as assessed by flow cytometry. Intracellular cytokine staining was after in vitro restimulation, transcription factor (TF) expression was assessed without in vitro restimulation. **c** Numbers of CD4+ T cells per LN and (**d**) frequencies of CD44+ cells in the CD4+ T cell population. Bar graphs show mean values ± SEM, each symbol corresponds to one mouse. For *n* values please refer to the legends to (**f**), (**i**), (**k**). **e** Contour plots showing IFNγ staining of CD4+ T cells from *Ms*-immunized mice. Data are concatenated from 5 mice. **f** Frequencies of total cytokine+ and Tbet+ CD4+ T cells or (**g**) IFNγ^hi^ (red gates in (**e**)) and Tbet^hi^ CD4+ T cells in *Ms*-immunized mice. **h**, **j** Contour plots showing IL-4 and IL-17A staining of CD4+ T cells from *Nb*- or *Ca-*immunized mice, respectively. Data are concatenated from 4 mice. **i**, **k** Frequencies of total cytokine+ and TF+ CD4+ T cells in *Nb-* or *Ca*-immunized mice. Cytokine graphs in (**f**, **g**) show mean ± SEM for groups of *n* = 9 (5 males + 4 females, PBS WT), 11 (6 males + 6 females, PBS KO), 10 (5 males + 5 females, *Ms* WT), and 12 (5 males + 7 females, *Ms* KO) mice over two independent experiments; the same *n* values also apply to the *Ms* bar graphs in (**c**, **d**). TF graphs show mean ± SEM for groups of *n* = 10 (PBS and *Ms* WT) or 9 (*Ms* KO) female mice over two independent experiments. Cytokine graphs in (**i**) show mean ± SEM for groups of *n* = 10 female mice over two independent experiments. The same *n* values also apply to the *Nb* bar graphs in (**c**, **d**). The TF graph shows mean ± SEM for *n* = 10 (PBS and *Nb* WT) or 8 (*Nb* KO) female mice examined over two independent experiments. Cytokine graphs in (**k**) show mean ± SEM for groups of *n* = 10 (PBS WT), 11 (PBS KO), 9 (*Ca* WT), or 14 (*Ca* KO) female mice over two independent experiments. The TF graph shows mean ± SEM for *n* = 8 (PBS WT and KO), 11 (WT *Ca*), or 10 (KO *Ca*) female mice over two independent experiments; the same *n* values also apply to the *Ca* bar graphs in (**c**, **d**). Statistical significance was assessed using a One-Way ANOVA with Holm-Sidak’s post-test (*Ms* and *Nb* TF) or Two-Way ANOVA with Sidak’s post-test (all remaining panels in **c**–**k**). Hash symbols refer to comparisons between PBS and immunized mice of the same genotype. Asterisks refer to comparisons between similarly immunized WT or CCR2-KO mice. NS: not significant; ^###^ *p* < 0.001; ^####^,****p < 0.0001. Exact values are shown for 0.05 > *p* > 0.01. Source data are provided as a Source Data file.

As monocytes were the most abundant Ag+ population in *Ms-* or *Ca*-immunized mice, we investigated CD4+ T cell responses in CCR2-KO mice which are devoid of circulating monocytes^29^ but harbor normal numbers of DC (Supplementary Fig. 5a). CCR2 deficiency led to a reduction in the number of *Ms*+ and *Ca*+, but not *Nb*+, cells in dLN, which was accounted for by an almost complete lack of Ag+ monocytes (Fig. 4a). Numbers of Ag+ DC in WT and CCR2-KO mice were similar, except for *Ms*+ CD11b^hi^ migDC2 which were reduced. In addition, *Ms* uptake per cell was significantly lower in CCR2-KO migDC2 compared to WT (Fig. 4b). A similar decrease was not observed in *Nb*+ migDC2 or *Ca*+ CD11b^hi^ migDC2, while *Ca* uptake by CD11b^low^ migDC2 was increased. Monocyte uptake in CCR2-KO hosts was not examined due to the low number of these cells. CD86 expression on PBS and Ag+ migDC2 was comparable regardless of genotype (Supplementary Fig. 5b).

In both CCR2-KO and WT mice, immunization with *Ms*, *Nb,* or *Ca* resulted in increased numbers of CD4+ cells in dLN on day 5, and increased percentages of CD44+ CD4+ T cells compared to PBS (Fig. 4c, d). However, frequencies of IFNγ+ and Tbet+CD4+ T cells were lower in CCR2-KO *Ms*-immunized mice compared to WT, with no compensatory enrichment in IL-4+ or IL-17A+ CD4+ T cells (Fig. 4e, f). The proportion of IFNγ-high CD4+ T cells, and their expression of Tbet, were also substantially decreased in CCR2-KO mice compared to WT, suggesting an impaired ability to differentiate into IFNγ+ effector T cells (Fig. 4g), which is consistent with previous reports on this mouse strain^29,30^. In contrast to the *Ms* response, CD4+ T cell responses to *Nb* and *Ca*, including IL-4+ and GATA-3+ responses to *Nb* as well as IL-17A+ and RORγt+ responses to *Ca*, were similar in CCR2-KO and WT mice (Fig. 4h–k), suggesting that the previously reported role of monocytes in amplifying Th2 immune responses to HDM^31^ or Th17 responses to oral *Ca* infection^32^ may not generally extend to other models of Th2 or Th17 immune responses.

CCR2 deficiency has been reported to affect the development of some migDC populations^33^. To establish whether the reduced IFNγ responses to *Ms* in CCR2-KO mice might be due to defects in DC responses, we used anti-GR1 treatment to deplete monocytes (as well as neutrophils) from WT mice starting 1 day before *Ms* immunization and throughout the experiment (Supplementary Fig. 5c, d). *Ms*+ monocytes in dLN were only partly depleted, whereas *Ms*+ migDC2 were unchanged (Supplementary Fig. 5e). CD86 expression by *Ms*+ migDC2 was increased following anti-GR1 treatment (Supplementary Fig. 5f). In line with this observation, LN cellularity and frequency of CD44+ CD4+ T cells on day 5 after *Ms* immunization were similar or higher in anti-GR1 treated mice compared to controls (Supplementary Fig. 5g). In agreement with the CCR2-KO data, the frequencies of IFNγ+, IFNγ-high, and Tbet+ CD44+ CD4+ T cells were significantly decreased by anti-GR1 (Supplementary Fig. 5h–k). However, given the increase of CD44+ CD4+ T cells following anti-GR1 treatment, these observations did not extend to cell numbers.

Together, these data indicate that monocytes are necessary to support the differentiation of IFNγ+ cells after *Ms* immunization, but are dispensable to the differentiation of IL-4+ or IL-17A+ CD4+ T cells to *Nb* or *Ca*.

### Monocyte-derived IL-12 is essential for sustained production of innate IFNγ in LN and the differentiation of IFNγ+ CD4+ T cells

**Fig. 5:**
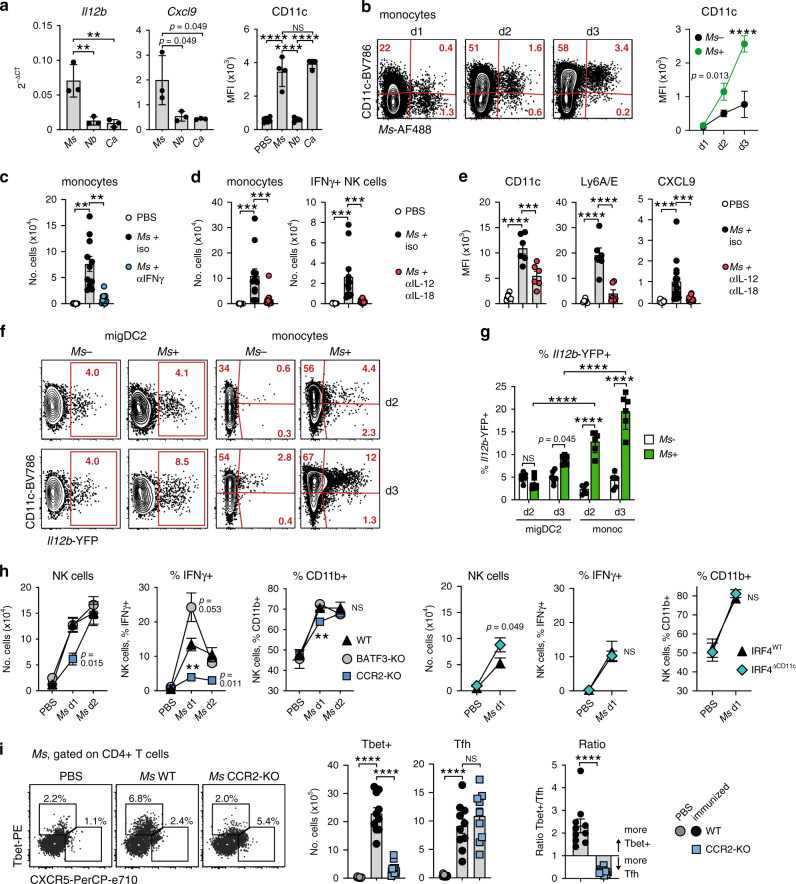
IL-12 from Ag+ monocytes drives early NK cell activation and IFNγ production to support Th1 responses in *Ms*-immunized mice. Mice of the indicated mouse strains were immunized i.d. with fluorescently labeled pathogens. LN cell suspensions were examined on day 2 after immunization unless otherwise indicated. **a** Expression of *Il12b* and *Cxcl9* transcripts and cell surface CD11c protein on total monocytes. RT-qPCR was on monocytes sorted from the pooled LN of five C57BL/6 female mice; each symbol refers to one biological replicate, *n* = 3. CD11c median fluorescence intensity (MFI) data are from groups of *n* = 6 (PBS) or four (immunized) female mice examined in one of five independent experiments that gave similar results; each symbol refers to one mouse. Bar graphs show mean ± SD. **b** Expression of CD11c on Ag− and Ag+ monocytes following *Ms* immunization. Contour plots show concatenated data from three mice, the line graph shows means ± SD for groups of *n* = 3 (*Ms* d1 and *Ms* d3) or 4 (*Ms* d2) female mice examined in one of two independent experiments that gave similar results. **c** Monocyte numbers in mice treated with neutralizing anti-IFNγ antibodies or isotype control. The bar graph shows mean ± SEM for groups of *n* = 10 (PBS) or 11 (*Ms*-immunized) female mice examined over two independent experiments; each symbol refers to one mouse. **d** Numbers of monocytes and IFNγ+ NK cells in mice treated with neutralizing anti-IL-12p40 + anti-IL-18 antibodies or isotype controls and immunized with *Ms*. Bar graphs show mean ± SEM for groups of *n* = 12 (monocytes) or 11 (NK cells) female mice examined over two independent experiments; each symbol refers to one mouse. **e** Expression of IFNγ-induced markers on monocytes from mice treated with neutralizing anti-IL-12p40 + anti-IL-18 antibodies or isotype controls and immunized with *Ms*. CD11c and Ly6A/E bar graphs show mean ± SEM for groups of *n* = 6 female mice from one of two independent experiments that gave similar results. CXCL9 bar graphs show mean ± SEM for groups of *n* = 11 (PBS) and 15 (*Ms*-immunized) female mice examined over 2 independent experiments. Symbols refers to individual mice. **f** Expression of *Il12b*-YFP in Ag− and Ag+ migDC2 and monocytes 2 and 3 days after immunization with CellTracker Orange-labeled *Ms*. Contour plots show concatenated data from 6 mice. **g** Frequencies of *Il12b*-YFP+ Ag− and Ag+ migDC2 and monocytes 2 and 3 days after *Ms* immunization. Bar graphs show mean ± SEM for groups of *n* = 6 male mice, each symbol refers to one mouse. Data are from one of three independent experiments that gave similar results. **h** Number of NK cells and frequency of IFNγ+ or CD11b+ NK cells in the indicated mouse strains 1 or 2 days after *Ms* immunization. Line graphs show mean ± SEM for groups of *n* = 10 (5 male + 5 female WT), 6 (3 male + 3 female BATF3-KO PBS), 8 (5 male + 3 female BATF3-KO *Ms* and CCR2-KO PBS), or 9 (CCR2-KO *Ms*) mice over two independent experiments, and 8 (5 male + 3 female IRF4^WT^ or IRF4^∆CD11c^ PBS) or 10 (5 male + 5 female IRF4^WT^ or IRF4^∆CD11c^ *Ms*) mice over two independent experiments. **i** Expression of Tbet and CXCR5 in CD4+ T cells from C57BL/6 (WT) or CCR2-KO mice 5 days after *Ms* immunization. Flow data are concatenated from five mice. Bar graphs show mean numbers±SEM for groups of *n* = 10 (PBS and *Ms* WT) or 9 (*Ms* KO) female mice tested over two independent experiments. Each symbol refers to one mouse. Statistical significance was assessed using One-Way ANOVA with Holm–Sidak’s post-test (**a**, **c**, **d**, **e**, **i** frequencies), two-Way ANOVA with Sidak’s post-test (**b**, **g**), mixed-effects ANOVA with Sidak’s post-test (**h**), or unpaired two-tailed Student’s *t* test (**i** ratio). NS: not significant, **p < 0.01, ***p < 0.001, ****p < 0.0001. Exact *p*-values are shown for 0.05 > *p* > 0.01. Source data are provided as a Source Data file.

To investigate the basis of the differential role of monocytes during immune responses to *Ms*, *Nb,* or *Ca*, we examined their phenotype during each type of response. On day 2 after immunization, total monocytes from *Ms*-immunized mice expressed increased levels of *Il12b* and the IFNγ-regulated transcript *Cxcl9* compared to monocytes from mice immunized with *Nb* or *Ca* (Fig. 5a). As the differentiation of monocytes into CD11c+ DC-like cells can also be regulated by IFNγ^34^, we assessed CD11c expression by monocytes after immunization and found that injection of *Ms* or *Ca*, but not *Nb*, induced CD11c upregulation (Fig. 5a, right panel). By simultaneously tracking CD11c expression and uptake of *Ms* antigen over time it was apparent that *Ms*+ monocytes preferentially acquired CD11c expression, with most *Ms*+ monocytes becoming CD11c+ by day 3 (Fig. 5b).

IFNγ is necessary for the release and maturation of inflammatory monocytes from the bone marrow^35^. Indeed, anti-IFNγ treatment significantly reduced monocyte numbers in LN on day 2 (Fig. 5c). Similarly, blockade of IL-12p40 and IL-18 reduced total monocyte numbers in LN, as well as IFNγ expression by LN NK cells, suggesting that IL-12p40 and IL-18 were key drivers of innate IFNγ production in this setting (Fig. 5d). Anti-IL-12p40 and anti-IL-18 treatment also reduced monocyte expression of IFNγ-induced markers including CD11c, Ly6A/E, and CXCL9 (Fig. 5e).

To determine the source of IL-12 in LN, we used *Il12b-*YFP reporter mice injected with *Ms*. The proportion of *Il12b-*YFP+ cell was higher in *Ms*+ monocytes compared to migDC2, and significantly enriched in the *Ms*+ CD11c+ monocyte population (Fig. 5f–g). To assess the relative contribution of DC and monocytes to IL-12 production, we investigated IFNγ production by NK cells in BATF3-KO mice, which lack the high *Il12b*-expressing XCR1+ migDC1 population, and in CCR2-KO and IRF4^∆CD11c^ mice. As shown in Fig. 5h, the number of NK cells in LN increased at a slower rate in CCR2-KO compared to WT and BATF3-KO mice but was comparable across groups on day 2. In contrast, the proportions of IFNγ+ NK cells in CCR2-KO mice were reduced at both time points despite expression of the NK cell activation marker CD11b^36^ being only marginally lower than WT on day 1 and unaffected on day 2. Proportions of IFNγ+ and CD11b+ NK cells were similar in IRF4^WT^ and IRF4^∆CD11c^ mice on day 1, and there was a trend to a higher IFNγ response in BATF3-KO mice on day 1 (*p* = 0.053). These results suggest that the IFNγ-dependent recruitment of monocytes to the LN is required to provide sufficient IL-12 to sustain innate IFNγ production.

Finally, we investigated in more depth the phenotype of the CD4+ T cell response to *Ms* in CCR2-KO mice. As shown in Fig. 5i, the reduced number of Tbet+ cells in CCR2-KO mice compared to WT was accompanied by an increase of BCL6+ CXCR5+ Tfh, leading to a reversal of the ratio of Tbet+/Tfh cells in CCR2-KO compared to WT mice. A similar finding was made in *Ms*-immunized animals treated with anti-GR1 (Supplementary Fig. 5j–l). Thus, monocytes are not essential for the priming of CD4+ T cell responses to *Ms* but provide key signals for their differentiation into Tbet+ IFNγ producers.

Together, these data show that, after *Ms* immunization, rapid IL-12p40- and IL-18-dependent IFNγ production attracts monocytes to the LN to provide essential mediators to sustain Th1 differentiation.

## Discussion

The aim of this study was to establish the APC requirements for CD4+ T cell differentiation in vivo. Studies in the literature currently provide support for two main models of Th differentiation: one in which the intrinsic properties of different APC are essential for appropriate Th differentiation^8^, and a second model proposing that such key properties are instead elicited by either innate signals triggered by pathogens^15^, or by the degree of activation of the APC itself^18^. As available evidence has been obtained using a variety of immune response models, ranging from live infection to in vitro immune responses, employing APC from different tissues, and measuring immune responses by different methods, it is probably unsurprising that the results from these disparate approaches do not easily integrate into a coherent model.

We sought to clarify the roles of APC subset specialization and context-dependent adaptation by carrying out a comparison of the APC requirements for CD4+ T cell differentiation within constant settings. We used skin-relevant inactivated pathogens injected into the same site in skin^19^, and monitored APC pathogen uptake and induction of immune responses in several models of APC deficiency. We chose inactivated pathogens as a more representative model of a physiological situation compared to proteins in adjuvant, and because they are able to provide a full range of innate signals to activate the immune response. Immune responses were studied in non-TCR transgenic mice, to exclude potential biases associated with the study of T cell populations of single specificity, and measured directly in dLN rather than indirectly through pathogen clearance or increased inflammation in tissues.

The results of this work led to several conclusions. Firstly, we observed that all pathogens, even when co-injected, were taken up by the same population of migDC2, suggesting no selective uptake of specific pathogens by different DC subsets. Within the heterogeneous migDC2 subset, uptake was similar regardless of the expression of CD11b or other markers reportedly associated with preferential recognition of Th2-inducing stimuli^23,37^. It is likely that the preferential uptake by migDC2 compared to migDC1 or LC was driven by either the favorable positioning of migDC2 with respect to the injection site, or by their superior ability to take up and present different types of antigens, as was also reported in other models^9,38,39^.

Secondly, we found that migDC2 were necessary for optimal Th responses to each of the pathogens used in this study. Decreased numbers of LN migDC2, as in IRF4^ΔCD11c^ mice^28^, led to decreased proportions of IFNγ-high CD4 + cells after *Ms* immunization, and mostly ablated IL-4+ and IL-17A+ CD4+ T cell responses in *Nb-* or *Ca*-immunized mice. T cell expression of the corresponding Th transcription factors Tbet, GATA-3 and RORγt reflected the cytokine findings, indicating that the responses being measured were not due to in vitro restimulation. The key role of migDC2 was further corroborated by the observation that defects in other migDC subsets, including migDC1 and LC, did not impair CD4+ T cell responses, and by the enrichment of immunization-induced transcriptional changes in Ag+ cells in this population^19,27^. These findings are partly consistent with previous observations that IRF4+ migDC2 are necessary for Th2 and Th17 immune responses in various tissues^9,10,23,37,40–42^ but, in contrast to some of those previous observations, also show that migDC2 are similarly necessary for differentiation of IFNγ+ CD4+ T cells, thereby supporting a model where the migDC2 population is not intrinsically programmed to induce a specific type of response but is essential for the differentiation of CD4+ Th cells of multiple phenotypes. Exposure to the appropriate cytokine conditioning is likely necessary for the expression of such properties^17,27^. It remains possible that this plasticity depends on the known heterogeneity within the migDC2 population^23,37,40^, or a requirement for IRF4 expression in other DC subsets. Lastly, the strong reduction in cytokine responses observed in IRF4^ΔCD11c^ vs. IRF4^WT^ mice was not paralleled by a comparable reduction in the numbers of CD4+ T cells in LN and their expression of CD44 after immunization, suggesting that CD4+ T cell priming and/or expansion may either require lower numbers of migDC2 compared to cytokine expression, or may rely on other APC populations in LN^22^.

**Fig. 6:**
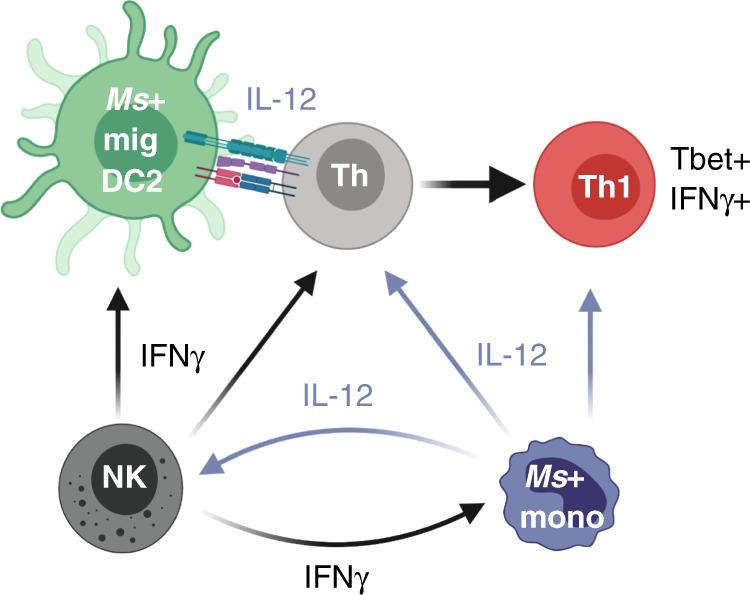
Monocytes are an essential source of IL-12 for NK cell activation. Diagram illustrating cytokine production by innate cell populations in LN during immunization with *Ms*. This figure was created with BioRender.com.

Our experiments also examined the requirement for monocytes. Unlike migDC2, inflammatory monocytes were required only in specific conditions. Expression of IFNγ and Tbet in CD4+ T cells from *Ms*-immunized CCR2-KO mice was substantially lower than in C57BL/6, whereas CD4+ T cell responses to *Nb* or *Ca* immunization were maintained. Several previous publications have reported that monocytes are required for effector Th1 differentiation^29,30,43,44^, although their role was not precisely defined in those studies. Here we show that monocytes were essential for NK cell production of IFNγ after *Ms* immunization. Together with the IL-12 and IL-18 dependency of NK-derived IFNγ, and higher *Il12b-*YFP reporter expression in monocytes compared to migDC2, this result suggests that monocytes were a critical source of IL-12 for innate IFNγ production (Fig. 6). In contrast, NK cell activation was intact in IRF4^ΔCD11c^ mice, which is consistent with our conclusion that the reduced IFNγ+ response in these mice was due to defective migDC2 migration rather than potential defects in the monocyte compartment. Collectively, these experiments suggest a co-operation between migDC2 and monocytes in which Ag+ migDC2 provide the high co-stimulation required for initial IL-12R expression on CD4+ T cells^45^, whereas monocytes enable production of high levels of innate IFNγ for Tbet upregulation^46^, IL-12R stabilization on CD4+ T cells^47^, as well as for the monocyte to moDC transition and their subsequent increased production of IL-12p40^34,48^. This proposed mechanism is consistent with data showing that IFNγ blockade and NK cell depletion^19^ or monocyte depletion^49^ both prevent the completion of the Th1 differentiation program while increasing the proportion of Tfh cells. Despite reduced IFNγ expression in *Ms*-immunized CCR2-KO mice, expression of other CD4+ T cell cytokines did not increase, suggesting that the progression of the Th1 developmental program was prevented without enabling alternative cytokine differentiation pathways^50^.

By using labeled pathogens, we were also able to evaluate the impact of APC mutations on both Ag uptake and expression of co-stimulatory molecules on Ag+ APC. IRF4^∆CD11c^ mice showed reduced numbers of total and Ag+ migDC2 in dLN with no compensatory increase in other Ag+ APC populations, possibly because migDC2 are still present in the skin of IRF4^∆CD11c^ mice^23,28^ where their Ag uptake is presumably normal. In contrast, Ag uptake by migDC2 was affected by CCR2 deficiency: the number of *Ms*+ CD11b^hi^ migDC2 was moderately decreased, and *Ms* uptake by both populations of migDC2 was lower than in WT mice, perhaps suggesting that monocytes facilitate Ag uptake and transport to LN. This possibility was also suggested by studies in CCR2-DTR mice treated with DT and infected with *Mycobacterium tuberculosis*^51^, although the substantial impact of DT treatment on DC2 numbers precludes clear conclusions from CCR2-DTR experiments. Our data revealed no clear correlation between CD86 expression on DC and impact on the resulting T cell response. For example, reduced CD86 expression on IRF4^∆CD11c^ *Ms*+ DC was not associated with a greater reduction in IFNγ response compared to CCR2-KO mice in which CD86 expression on *Ms*+ DC was intact. These results suggest that factors other than CD86 play a more prominent role in determining the outcome of the T cell response to *Ms*.

Finally, it is important to note that our experiments focused on cytokine production and primary immune responses at a single timepoint, without assessing the kinetics of the CD4+ T cell response or memory development. The APC and co-stimulatory or cytokine requirements for those responses may differ from those of the initial cytokine+ CD4+ T cell pool.

In conclusion, we show that uptake of labeled inactivated pathogens identifies a population of migDC2 that is necessary for the differentiation of CD4+ T cells expressing IFNγ, IL-4, or IL-17A, and identify a key role of monocytes in supporting early innate IFNγ production for Th1 differentiation. We provide evidence that migDC2 respond to different pathogens by expressing similar levels of co-stimulatory molecules such as CD40 and CD86, but different chemokine and cytokine transcripts, suggesting that these cytokines and chemokines are essential factors in migDC2 instruction of CD4+ Th cell differentiation. It will be interesting to determine whether a similar level of plasticity is also observed in DC2 from tissues other than skin, or whether further studies will reveal migDC2 heterogeneity or functional specializations that are not correlated to the surface markers used in this study.

## Methods

### Mice

Mice were bred and housed under specific pathogen-free conditions at the Malaghan Institute of Medical Research (ambient temperature 21 ± 3 °C, humidity 50 ± 10%, light/dark cycle 12/12 h). All mice were between 6 and 14 weeks of age and were age- and sex-matched within experiments. C57BL/6J (stock no. 000664), CCR2-KO (stock no. 004999), *Il12b*-YFP (stock no. 006412), *Itgax*-cre (stock no. 008068) and *Irf4*^fl^ (stock no. 009380) breeders were originally obtained from the Jackson Laboratory (Bar Harbor, ME); BATF3-KO^52^ and Langerin-DTR^53^ breeders were from Dr. Kenneth Murphy (Washington University, St Louis, MO) and Dr. Bernard Malissen (Centre d’Immunologie de Marseille-Luminy, Marseille, France), respectively. All experimental mice were housed in the same room and caged separately by strain. *Itgax*-cre^+/−^ *Irf4*^fl/fl or fl/−^ (IRF4^ΔCD11c^) and *Itgax*-cre^−/−^ *Irf4*^fl/fl or fl/−^ (IRF4^WT^) mice were generated by crossing *Itgax*-cre to *Irf4*^fl^ for two generations and were co-housed from birth. For some experiments, CCR2-KO mice (JAX stock no. 004999) were obtained from the NIAID contract facility at Taconic Biosciences and C57BL/6Tac control mice were obtained from Taconic Biosciences (Rensselaer, NY); both strains were housed at an AALAC accredited animal facility at the NIAID, NIH (ambient temperature 22 ± 3 °C, humidity 50 ± 20%, light/dark cycle 14/10 h) in separate adjacent caging. Mice were euthanized by CO_2_ inhalation or cervical dislocation. All experimental protocols were approved by the Victoria University of Wellington Animal Ethics Committee (protocol reference: 23907) or the NIAID Animal Care and Use Committee (protocol reference: LPD72) and were performed in accordance with institutional guidelines.

### Bone marrow chimeras

IRF4^ΔCD11c^ and IRF4^WT^ donor mice^41^ were bred at the University of Manchester Biological Services Facility from breeding pairs provided by Dr. William Agace (Lund University, Sweden). Bone marrow cell suspensions from IRF4^ΔCD11c^ or IRF4^WT^ mice were used to reconstitute lethally irradiated (two doses of 500cGy) C57BL/6J recipients by intravenous injection. After reconstitution, chimeras were rested for 10+ weeks before immunization.

### Preparation of immunogens

*M. smegmatis* (*Ms*, mc2155) was grown in Luria-Bertani broth overnight at 37 °C. Bacteria were washed thrice in PBS containing 0.05% Tween 80 and heat-killed at 75 °C for 1 h. *N. brasiliensis* (*Nb*) infective L3 larvae were prepared, washed in sterile PBS and killed by three freeze-thaw cycles as described^54^. *C. albicans* (*Ca*, ATCC10321) was kindly provided by Prof. Richard Cannon (University of Otago, New Zealand). Yeasts were propagated by inoculating sterile yeast extract-peptone-dextrose broth and incubating the culture under agitation at 30 °C for 72 h. Yeast was washed thrice in PBS and heat-killed at 75 °C for 1 h.

For fluorescent labeling of pathogens, non-viable *Ms, Nb,* or *Ca* were incubated in 0.05 M NaHCO_3_ buffer and 0.1 mg AF488 NHS Ester (Molecular Probes, Invitrogen) and then washed with 0.1 M Tris buffer. For the pathogen mixing experiments in Fig. 1, and experiments in IRF4^ΔCD11c^ mice which express GFP in CD11c+ cells, pathogens were labeled with CellTracker™ Orange CMTMR (CTO, Invitrogen™, ThermoFisher Scientific, MA) or Cell Proliferation Dye eFluor670 (eBioscience, ThermoFisher Scientific, MA) according to the manufacturer’s instructions.

### Immunizations and in vivo treatments

For immunizations, mice were anesthetized and injected intradermally (i.d.) with 4 × 10^6^ CFU heat-killed *Ms*, 300 non-viable *Nb*, 1 × 10^7^ heat-killed *Ca* or PBS in a 30μl volume into the ear pinna. Pathogen doses that induce optimal CD4 + Th cell cytokine responses were determined in dose–response experiments as described^19^.

To deplete DTR expressing cells, Langerin-DTR mice were treated with 20 ng/g diphtheria toxin (DT; Sigma-Aldrich) two days before immunization.

For in vivo cytokine neutralization, mice were injected with 500 µg anti-IL-12p40 (C17.8), 300 µg anti-IL-18 (YIGIF74-1G7), 500 µg anti-IFNγ (XMG1.2), or 500 µg of the appropriate isotype control (2A3 or HPRN) 2 h before immunization. Animals received a second dose of antibody one day later and were euthanised on day 2. All antibodies were from BioXCell (West Lebanon, NH).

### Cell preparations

For assessment of T cell intracellular cytokines, LN single-cell suspensions were prepared by gently pressing LN through a 70 µm cell strainer, washed with IMDM, and cultured in fetal calf serum-supplemented IMDM (Invitrogen) in the presence of 50 ng/mL PMA (Sigma-Aldrich), 1 µg/mL ionomycin (Merck Millipore) and 1 µL/mL GolgiStop™ (BD Pharmingen) for 5 h at 37 °C.

DC were prepared from auricular LN by digesting with 100 µg/mL Liberase TL and 100 µg/mL DNase I (both from Roche, Germany) for 25 min at 37 °C before passing through a 70 µm cell strainer. For assessment of cytokine production by DC, monocytes, and NK cells, single-cell suspensions were incubated in fetal calf serum-supplemented IMDM in the presence of GolgiStop™ (BD Pharmingen) and Brefeldin A (Sigma Aldrich) for 6 h.

### Flow cytometry

For staining of cell surface molecules, cells were suspended in anti-mouse CD16/32 (clone 2.4G2, affinity purified from hybridoma culture supernatant) to block Fc receptors prior to labeling with cocktails of fluorescent antibodies diluted in PBS containing 2 mM EDTA, 0.01% sodium azide and 2% FCS. Antibodies specific for murine BCL6 (clone K112-91), CD3 (145-2C11), CD4 (RM4-5 and GK1.5), CD8 (53-6.7), CD11b (M1/70), CD11c (HL3), CD40 (3/23), CD44 (IM7), CD45R (B220, clone RA3-6B2), CD86 (GL-1), CD103 (M290), CD185 (CXCR5, clone 2G8), CD273 (PDL2, clone TY25), CD326 (G8.8), Ly6A/E (Sca1, clone D7), Ly6C (AL-21), Ly6G (1A8), MHCII (M5/114.15.2), NK1.1 (PK136), TCRβ (H57-597) together with Streptavidin conjugates were from BD Biosciences. Antibodies specific for murine CD3 (clone 145-2C11), CD11c (N418), CD19 (6D5), CD64 (X54-5/7.1), CD169 (3D6.112), CD197 (CCR7, clone 4B12), CD206 (Mannose receptor, clone C068C2), CD279 (PD1, clone 29 F.1A12), CD301b (MGL2, clone URA-1), CD326 (G8.8), Ly6C (HK1.4), Ly6G (1A8), MHCII (M5/114.15.2), TCRβ (H57-597), T-bet (4B10), XCR1 (ZET), and the Zombie NIR^TM^ Fixable Viability Kit were from BioLegend. Antibodies specific for murine CD4 (clone GK1.5), CD19 (eBio1D3), CD44 (IM7), CD185 (CXCR5, clone SPRCL5), as well as DAPI (4’,6-Diamidino-2-Phenylindole, Dihydrochloride): Live Dead® Fixable Aqua and Live Dead® Fixable Blue were from ThermoFisher Scientific. Anti-MHCII (3JP / Y-3P^55^) anti-CD4 (GK1.5^56^) and anti-CD8 (2.43^57^) were prepared in-house, labeled with AF488 or AF647 according to the manufacturer’s instructions (Molecular Probes, Invitrogen) and used at 1:200 dilution. Prior to running samples, cells were stained with DAPI (Molecular Probes, Invitrogen) to identify dead cells.

For intracellular cytokine staining, cells were stained with LIVE/DEAD® Fixable Blue (ThermoFisher Scientific) prior to staining of cell surface molecules. Cells were then fixed and permeabilized using a BD Cytofix/Cytoperm kit (BD Pharmingen) and stained for intracellular cytokines. Anti-IL-4 (11B11) and anti-IFNγ (XMG1.2) were from BD Biosciences or BioLegend, anti-IL-17A was from ThermoFisher (eBio17B7), and anti-CXCL9 (MIG-2F5.5) was from BioLegend.

For staining of transcription factors, cells were stained with LIVE/DEAD® Fixable Blue prior to staining of cell surface molecules. Cells were then fixed and permeabilized using a TrueNuclear^™^ Transcription Buffer set (BioLegend) prior to staining with anti-BCL6 (K112-91; BD Horizon), anti-GATA-3 (L50-823; BD Biosciences), anti-RORγt (Q31-378; BD Biosciences), or anti-Tbet (4B10; BioLegend).

For staining of chemokine receptors, cells were incubated with anti-CXCR5 (clone 2G8; BD Pharmingen) or anti-CD197 (CCR7; clone 4B12; BioLegend) for 30 min at 37 °C prior to staining for additional cell surface antigens.

All samples were collected on a LSRFortessa SORP^™^, LSRII SORP^™^, FACSymphony A5 SORP^™^ flow cytometer (all from Becton Dickinson) using BD FACSDiva^TM^ version 6.1.1, 8.0.2 and 1.4 respectively, or an Aurora spectral cytometer (Cytek eBiosciences) using SpectroFlo version 2.2.0. Flow data were analyzed using the FlowJo software version 10.7.1 (Treestar Inc). Compensation was set in each experiment using UltraComp eBeads^™^ (eBioscience) and dead cells and doublets were excluded from analysis.

### Cell sorting

DC were enriched from single-cell suspensions by negative selection using a Dynabead^®^ Mouse DC Enrichment kit (Invitrogen™, ThermoFisher Scientific) as per the manufacturer’s instructions. Enriched DC were stained with fluorescently labeled antibodies including: anti-CD11b (M1/70; BD Pharmingen), anti-CD11c (HL3; BD Horizon), anti-IA/IE (MHCII; M5/114; BD Horizon), anti-XCR1 (ZET; BioLegend), anti-CD326 (G8.8; BioLegend), anti-Ly6C (HK1.4; BioLegend), anti-Ly6G (1A8; BD Horizon), anti-CD45R (B220; RA3-6B2; BD Horizon) and anti-CD3 (145-2C11), then sorted into total monocytes, Ag− XCR1+ DC1, Ag− CD11b^hi^ DC2, Ag+ CD11b^hi^ DC2, Ag− CD11b^low^ DC2 and Ag+ CD11b^low^ DC2 subsets using a BD Influx (Becton Dickinson) using BD FACS^TM^ version 1.2.0.142 software. For each subset, 2000 cells were collected.

### RNA extraction and qRT-PCR on sorted DC subsets

RNA was extracted from sorted DC populations using a Quick-RNA™ MicroPrep kit (Zymo Research) and converted to cDNA using a High Capacity RNA-to-cDNA kit (Applied Biosystems, ThermoFisher Scientific) according to the manufacturer’s directions. cDNA was pre-amplified using a Sso-Advanced™ PreAmp Supermix kit (Bio-Rad) before running qRT-PCR, which was performed using the TaqMan™ Gene Assay platform (Applied Biosystems, ThermoFisher Scientific). The TaqMan™ probes used in this study were: *Il12a* (Mm00434169_m1), *Il12b* (Mm01288989_m1), *Il27a* (Mm01313472_m1), *Ebi3* (Mm00469294_m1), *Cxcl9* (Mm00434946_m1), *Ccl17* (Mm01244826_g1), *Ccl22* (Mm00436439_m1), *Tnfsf4* (Mm00437214_m1), *Il23a* (Mm00518984_m1) and *Il6* (Mm00446190_m1).

### Statistical analyses

Statistical analyses were performed using Prism 8.0 GraphPad software. Data were analyzed using an un/paired two-sided Student’s *t* test, One-Way ANOVA with Holm–Sidak’s post-test, Two-Way ANOVA with Sidak’s multiple comparisons test, or as indicated in Figure legends. *p*-values < 0.05 were considered statistically significant and are reported in figures using the following notation: NS: not significant; **^,##^*p* < 0.01; ***^,###^*p* < 0.001; ****^,####^*p* < 0.0001 (with * and # referring to the comparisons specified in each Figure legend). Exact values are shown for 0.05 > *p* > 0.01.

### Reporting summary

Further information on research design is available in the Nature Research Reporting Summary linked to this article.

## Supplementary information

Supplementary Information

Reporting Summary

## Data Availability

The authors declare that the data supporting the findings of this study are available within the paper and its supplementary information files or are available from the authors upon reasonable requests. Source data are provided with this paper.

## Source data

Source Data
