# Supplementary material for: Dermal IRF4+ dendritic cells and monocytes license CD4+ T helper cells to distinct cytokine profiles
Source: Nat Commun. 2020 Nov 6;11:5637. doi: 10.1038/s41467-020-19463-9 (PMC7647995; doi:10.1038/s41467-020-19463-9)
Supplement: Supplementary file 1 — Supplementary Information [file 41467_2020_19463_MOESM1_ESM.pdf]

Dermal IRF4+ dendritic cells and monocytes prime CD4+ T helper cells to distinct cytokine profiles

Supplementary information

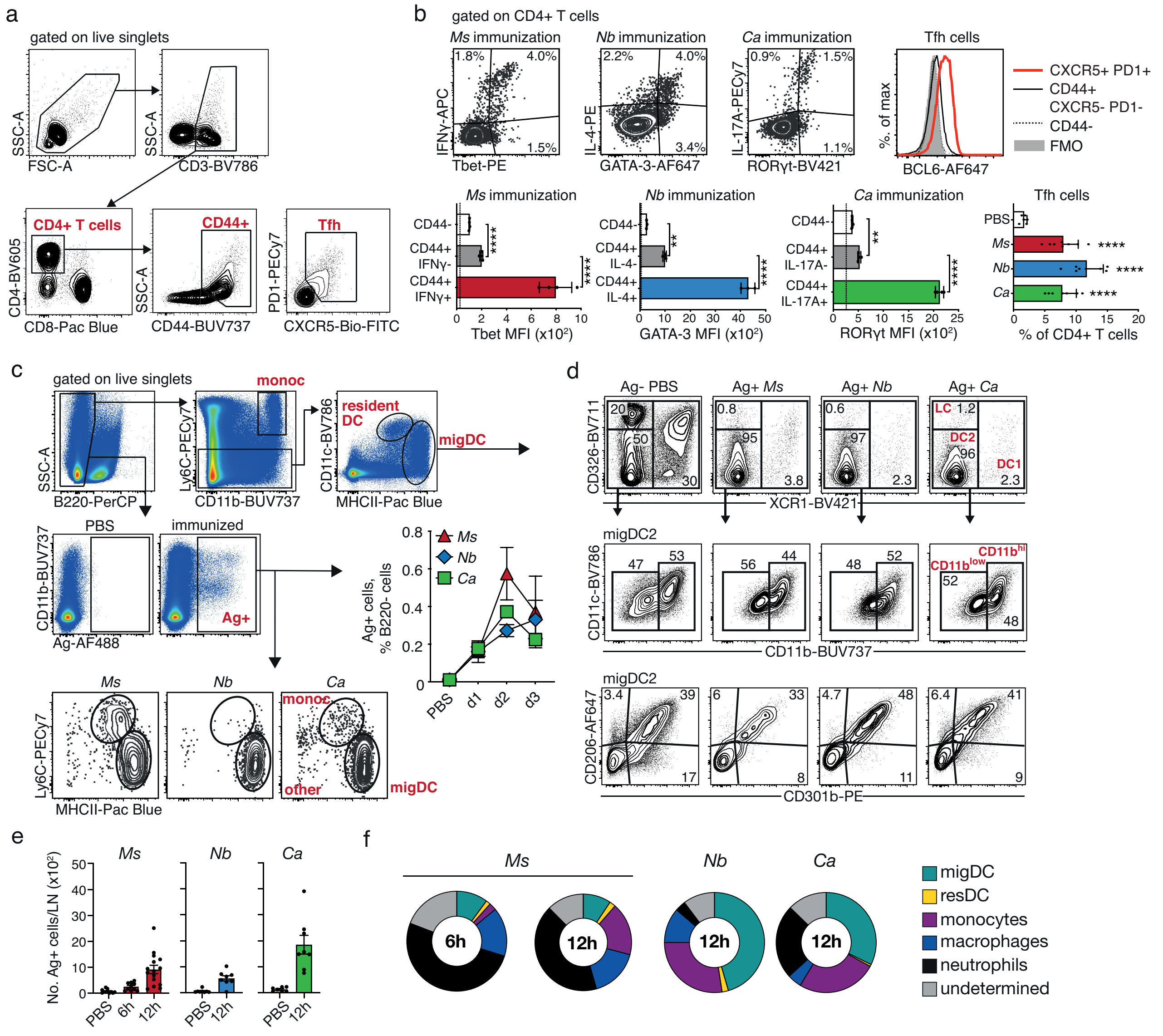

Supplementary Fig. 1: Antigen (Ag) uptake and CD4+ T cell responses after immunization with *Ms*, *Nb* or *Ca*.

Female C57BL/6 mice were immunized i.d. with *Ms*, *Nb*, *Ca*, or PBS as a control. Flow cytometry analysis was performed on lymph node (LN) cell suspensions at the indicated time points.

(a) Gating strategy for identifying CD4+ T cells in skin-draining LN. Gating is related to Fig. 3c-k, Fig. 4c-k, Fig. 5i, Supplementary Fig. 1b, Supplementary Fig. 4b-g, and Supplementary Fig. 5g-l.

(b) Cytokine and transcription factor (TF) expression in CD4+ T cells 5 days after immunization. LN cell suspensions were either re-stimulated with PMA/ionomycin and examined for cytokine and TF expression by intracellular flow cytometry or examined for expression of surface CXCR5 and PD1 and intracellular BCL6 without re-stimulation to identify follicular T helpers (Tfh). The dotted lines in the bar graphs refer to the median fluorescence intensity (MFI) of isotype staining controls. TF bar graphs show mean ± SD for groups of n = 3 (*Nb*) or 4 (*Ms* and *Ca*) mice from 1 of 2 independent experiments that gave similar results. The Tfh bar graphs show mean ± SD for groups of n = 6 (PBS), 7 (*Ca*) or 8 (*Ms* and *Nb*) mice over 2 independent experiments. Each dot corresponds to one mouse. Statistical significance was assessed using One-Way ANOVA with Holm-Sidak's post-test. **p<0.01, **** p<0.0001.

(c) Gating strategy for identifying Ag+ DC and monocytes. Gating is related to Fig. 1a-i, Fig. 2a-d, Fig. 3a-b, Fig. 4a-b, Fig. 5b, f, g, Supplementary Fig 1d-f, Supplementary Fig. 3a-c, Supplementary Fig. 4a, and Supplementary Fig 5a-b, d-f. Frequencies of Ag+ cells 1-3 days after immunization with AF488-labeled *Ms*, *Nb* or *Ca* are shown in the line graph. Symbols show mean ± SEM for groups of n = 6 (*Ms* d1, *Ms* d3, *Nb* d3,

Ca d3), 9 (*Nb* d1, *Ca* d1), 11 (*Ms* d2), 13 (*Nb* d2, *Ca* d2) or 14 (PBS) mice from 3 (*Ms*) or 4 (*Nb* and *Ca*) independent experiments.

(d) Gating strategy to assess the distribution of total and Ag+ cells in migratory DC (migDC) and migDC2 subsets in LN as in Fig. 1d and 1e, respectively. Contour plots show concatenated data from either 3 (PBS, two top panels) or 5 (lower PBS panel and all immunized groups) mice.

(e) Total numbers of Ag+ cells in LN at different times after immunization. Bar graphs show mean ± SEM for groups of n = 8 (PBS, *Nb* and *Ca*), 10 (*Ms* 6h) or 15 (*Ms* 12h) mice examined over 2 (*Ms* 6h, *Nb* and *Ca*) or 3 (*Ms* 12h) independent experiments.

(f) Cellular composition of Ag+ populations in LN at 6h or 12h after immunization. Data refer to n = 5 mice/condition from 1 of 2 independent experiments that gave similar results. Source data are provided as a Source Data file.

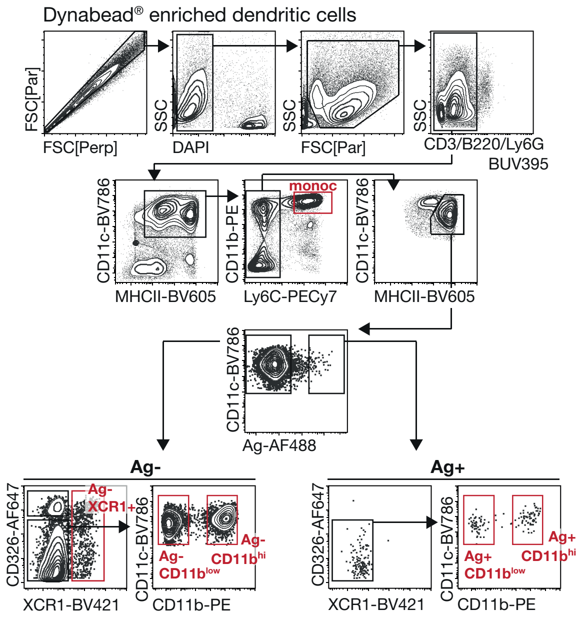

Supplementary Fig. 2: Gating strategy for sorting Antigen (Ag)+ DC.

Female C57BL/6 mice were immunized i.d. with *Ms*, *Nb*, *Ca*, or PBS as a control. Lymph node cell suspensions were prepared two days after immunization. Contour plots show the gating strategy used to sort Dynabead-enriched Ag+ and Ag- DC and total monocyte populations for the RT-qPCR analyses in Fig. 2e and Fig. 5a.

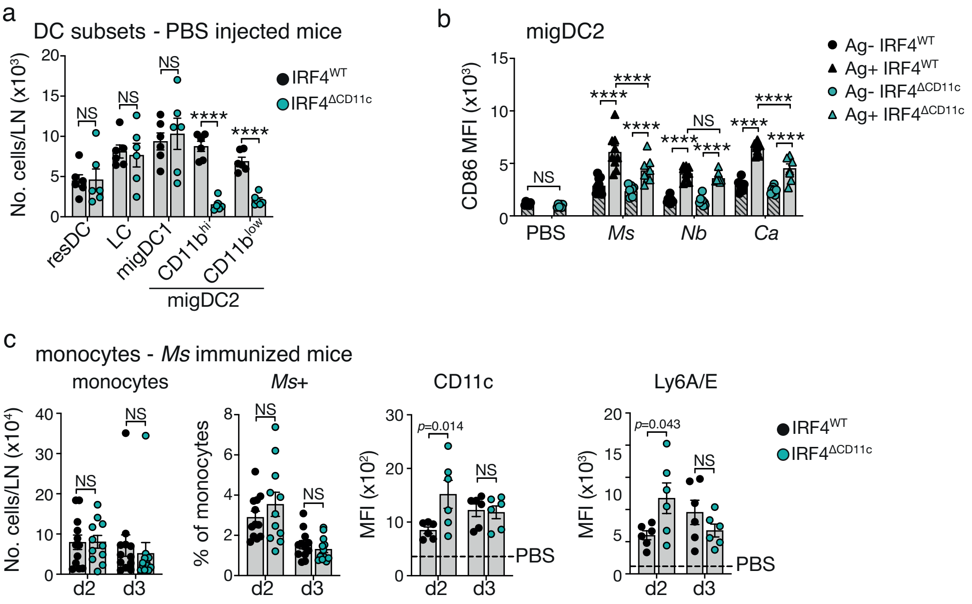

Supplementary Fig. 3: Impact of conditional IRF4 deletion on DC and monocyte numbers and activation.

Mice were immunized i.d. with *Ms*, *Nb*, *Ca*, or PBS as a control. Lymph node (LN) cell suspensions were examined by flow cytometry at the indicated time points. Each symbol corresponds to one mouse.

(a) DC numbers in PBS-treated $IRF4^{WT}$→ or $IRF4^{\Delta CD11c}$→ female C57BL/6 bone marrow chimeras.

(b) CD86 expression on Antigen (Ag)- and Ag+ migDC2 in $IRF4^{WT}$→ or $IRF4^{\Delta CD11c}$→C57BL/6 bone marrow chimeras 2 days after immunization. Bar graphs show mean ± SEM for groups of n = 6 (all PBS $IRF4^{WT}$ and $IRF4^{\Delta CD11c}$), 8 (*Ca* $IRF4^{WT}$, all immunized $IRF4^{\Delta CD11c}$) or 9 (*Ms* $IRF4^{WT}$ and *Nb* $IRF4^{WT}$) female chimeras over 2 independent experiments.

(c) Number of total monocytes, frequency of *Ms*+ monocytes and expression of CD11c and Ly6A/E in $IRF4^{WT}$ or $IRF4^{\Delta CD11c}$ monocytes on day 2 or 3 after *Ms* immunization. Bar graphs show mean ± SEM for groups of n = 12 (6 male + 6 female; number and frequency bar graphs) mice from 2 independent experiments, or n = 6 (CD11c and Ly6A/E expression) female mice from 1 of 2 independent experiments that gave similar results.

Statistical analysis was by unpaired two-tailed Student's t-test (a) or Two-Way ANOVA with Sidak's post-test (b, c). NS: not significant; **** $p<0.0001$. Exact p-values are shown for $0.05>p>0.01$. Source data are provided as a Source Data file.

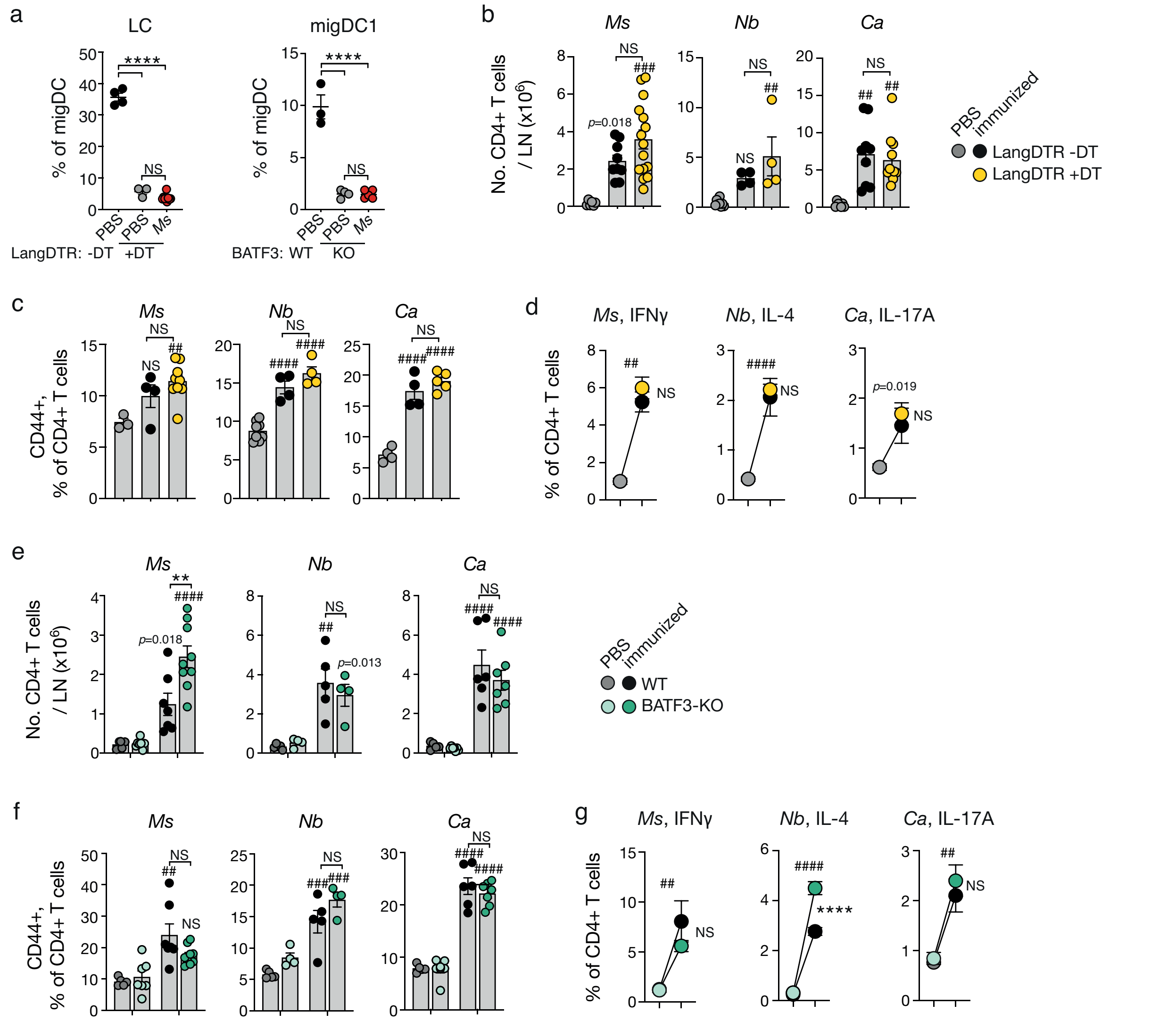

Supplementary Fig. 4: Langerhans cells (LC) and migratory (mig)DC1 are not required for CD4+ T cell cytokine production after *Ms*, *Nb* or *Ca* immunization.

LangDTR mice that were treated with diphtheria toxin (DT) or vehicle and BATF3-KO mice were immunized i.d. with *Ms*, *Nb*, *Ca*, or PBS as a control. CD4+ T cell responses were examined in lymph node (LN) cell suspensions by flow cytometry 5-7 days after immunization.

(a) Frequencies of LC in LangDTR mice, and migDC1 in BATF3-KO mice. Bar graphs show mean± SEM for groups of n = 4 (LangDTR no DT PBS and BATF3-KO PBS), 3 (LangDTR+DT PBS and WT PBS), 9 (LangDTR+DT *Ms*) or 5 (BATF3-KO *Ms*) female mice from one of two independent experiments that gave similar results. Each symbol refers to one mouse.

(b-d) CD4+ T cell responses to *Ms*, *Nb* or *Ca* in LangDTR mice.

(b) Number of CD4+ T cells per LN across immunizations. Bar graphs show mean ± SEM, group sizes are as in (d). Each symbol refers to one mouse.

(c) Frequencies of CD44+ cells in the CD4+ T cell population. Bar graphs show mean ± SEM for groups of n = 3, 4 and 9 (PBS no DT, *Ms* no DT and *Ms* + DT, respectively); 8, 4 and 4 (PBS no DT, *Nb* no DT and *Nb* + DT, respectively), and 4, 4, and 5 (PBS no DT, *Ca* no DT and *Ca* + DT, respectively) female mice from one of two independent experiments that gave similar results. Each symbol refers to one mouse.

(d) Frequencies of cytokine+ CD4+ T cells in immunized mice. Graphs show mean ± SEM for groups of n = 6, 9 and 15 (PBS no DT, *Ms* no DT and *Ms* + DT, respectively) and 7, 9, and 11 (PBS no DT, *Ca* no DT and *Ca* + DT,

respectively) female mice from two independent experiments, or *n* = 8, 4 and 4 (PBS no DT, *Nb* no DT and *Nb* + DT, respectively) female mice from one of two independent experiments that gave similar results.

(e-g) CD4+ T cell responses to *Ms*, *Nb* or *Ca* in BATF3-KO and C57BL/6 control mice.

(e) Number of CD4+ T cells per LN across immunizations.

(f) Frequencies of CD44+ cells in the CD4+ T cell population.

(g) Frequencies of cytokine+ CD4+ T cells in immunized mice.

Graphs in (e-g) show mean ± SEM for groups of *n* = 5, 7, 7 and 9 (WT PBS and *Ms*; KO PBS and *Ms*, respectively) and 5, 6, 7, and 7 (WT PBS and *Ca*; KO PBS and *Ca*, respectively) female mice from two independent experiments, or *n* = 5, 5, 4 and 4 (WT PBS and *Nb*; KO PBS and *Nb*, respectively) female mice from one of two independent experiments that gave similar results. In (e), (f) each symbol refers to one mouse.

Statistical significance was assessed using One-Way ANOVA with Holm-Sidak's post-test (a-d) or Two-Way ANOVA with Sidak's post-test (e-g). NS: not significant; ##,**p<0.01; ###p<0.001; ####,**** p<0.0001. Hash symbols refer to comparisons between PBS and immunized mice of the same genotype. Asterisks refer to comparisons between similarly immunized mice of different genotypes. Exact p-values are shown for 0.05>p>0.01. Source data are provided as a Source Data file.

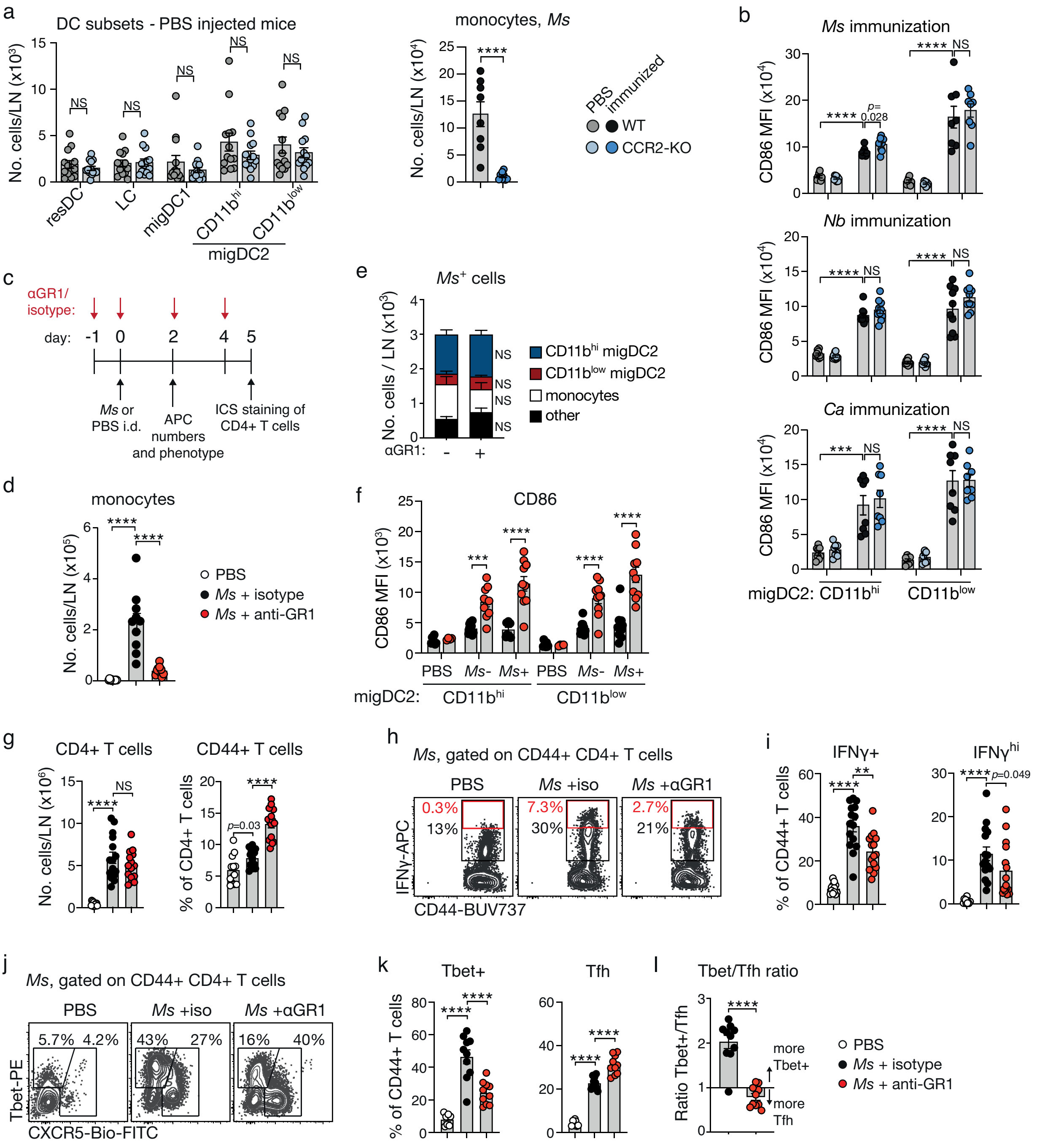

Supplementary Fig. 5: Monocyte depletion differentially affects the development of Tbet+ and T follicular helper (Tfh) CD4+ T cells after *Ms* immunization.

The role of monocytes in *Ms* immune responses was examined in CCR2-KO mice and C57BL/6 mice treated with anti-GR1. Mice were immunized i.d. with *Ms* or PBS as a control, and immune responses were measured in lymph node (LN) cell suspensions at the indicated times. Bar graphs show mean ± SEM; symbols refer to individual mice (except (e).

(a) DC numbers in PBS-injected CCR2-KO and WT mice. Monocyte numbers were determined two days after *Ms* immunization. Group sizes for the DC bar graphs are n = 13 (4 female + 9 male) mice over 3 independent experiments. Group sizes for the monocyte bar graph are n = 8 (4 female + 4 male) mice over 2 independent experiments.

(b) CD86 expression on $CD11b^{hi}$ and $CD11b^{low}$ migDC2 from CCR2-KO and WT mice, two days after PBS, *Ms*, *Nb* or *Ca* injection. MFI: median fluorescence intensity. Group sizes are *n* = 8 (4 female + 4 male, *Ms*), 10 male (*Nb*) and 8 male (*Ca*) mice, each examined over two independent experiments.

(c) Experimental timeline of anti-GR1 treatments, immunizations and analyses for the experiments in (d-l).

(d) Number of monocytes in the LN of aGR1-treated mice 2 days after *Ms* immunization. Group sizes are *n* = 12 (PBS+isotype) or 10 (*Ms*) female mice over two independent experiments.

(e) Cellular composition of Ag+ populations from isotype and anti-GR1-treated mice. Group sizes are *n* = 10 female mice over two independent experiments.

(f) CD86 expression on Ag- and Ag+ migDC2 subsets from isotype and anti-GR1-treated mice. Group sizes are *n* = 8 (isotype PBS), 4 (anti-GR1 PBS) and 10 (all *Ms*- and *Ms*+) female mice examined over two independent experiments.

(g-l) CD4+ T cell responses to *Ms* in isotype and anti-GR1 treated mice were measured by intracellular cytokine staining following PMA/ionomycin re-stimulation, or by assessing the expression of Tbet and Tfh markers without re-stimulation.

(g) Number of total CD4+ T cells and frequency of CD44+ cells in the CD4+ T cell population. Group sizes are *n* = 12 (PBS) and 15 (*Ms* ± aGR1) female mice examined over 3 independent experiments.

(h) Contour plots showing IFNγ expression in CD44+ CD4+ T cells. Data are concatenated from 5 mice/group; black and red gates highlight the IFNγ+ and IFNγhi populations respectively.

(i) Frequencies of IFNγ+ and $IFN\gamma^{hi}$ CD4+ T cells in *Ms*-immunized mice. Group sizes are as in (g).

(j) Contour plots showing Tbet and CXCR5 expression in CD44+ CD4+ T cells concatenated from 4 (PBS) or 5 (*Ms*-immunized) mice/group.

(k) Frequencies of Tbet+ CD4+ T cells and CXCR5+PD1+ Tfh cells in *Ms*-immunized mice. Group sizes are *n* = 7 (PBS) and 10 (*Ms* ± aGR1) female mice examined over two independent experiments.

(l) Ratio of Tbet+ / Tfh cells in LN from isotype and anti-GR1-treated mice immunized with *Ms*. Group sizes are *n* = 10 ratios from 10 female mice examined over two independent experiments.

Statistical significance was assessed using a Two-Way ANOVA with Sidak's post-test (DC in a, panels b and f), One-Way ANOVA with Holm-Sidak's post-test (d, g, i, k), and an unpaired two-sided Student's t-test (monocytes in a, panels e and l). NS: not significant, **p<0.01, ***p<0.001, **** p<0.0001. Exact p-values are shown for 0.05>p>0.01. Source data are provided as a Source Data file.

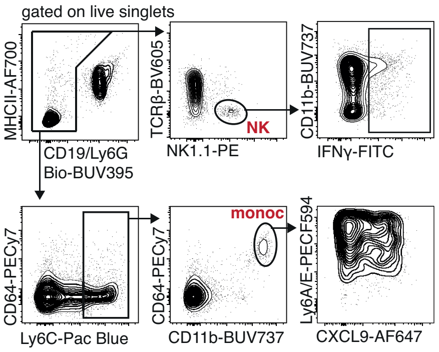

Supplementary Fig. 6: Gating strategy for NK cells and monocytes in *Ms*-immunized mice

Lymph node cell suspensions were prepared from *Ms*-immunized mice. Intracellular staining for IFNγ+ NK cells and CXCL9+ monocytes was after 5h incubation in brefeldin A and BD GolgiStop™ before fixation and permeabilization. Data refer to a C57BL/6 female mouse immunized with *Ms* 2 days previously. The gating shown refers to panels 5c, 5e (monocytes) and 5d, 5h (NK cells).
