# Supplementary material for: Dermal IRF4+ dendritic cells and monocytes license CD4+ T helper cells to distinct cytokine profiles
Source: Nat Commun. 2020 Nov 6;11:5637. doi: 10.1038/s41467-020-19463-9 (PMC7647995; doi:10.1038/s41467-020-19463-9)
Supplement: Supplementary file 2 — Reporting Summary [file 41467_2020_19463_MOESM2_ESM.pdf]

nature research

Corresponding author(s): Franca Ronchese

Last updated by author(s): 2020 09 29

Reporting Summary

Nature Research wishes to improve the reproducibility of the work that we publish. This form provides structure for consistency and transparency in reporting. For further information on Nature Research policies, see our Editorial Policies and the Editorial Policy Checklist.

Statistics

For all statistical analyses, confirm that the following items are present in the figure legend, table legend, main text, or Methods section.

n/a	Confirmed	
[ ]	[x]	The exact sample size (*n*) for each experimental group/condition, given as a discrete number and unit of measurement
[ ]	[x]	A statement on whether measurements were taken from distinct samples or whether the same sample was measured repeatedly
[ ]	[x]	The statistical test(s) used AND whether they are one- or two-sided<br>*Only common tests should be described solely by name; describe more complex techniques in the Methods section.*
[x]	[ ]	A description of all covariates tested
[ ]	[x]	A description of any assumptions or corrections, such as tests of normality and adjustment for multiple comparisons
[ ]	[x]	A full description of the statistical parameters including central tendency (e.g. means) or other basic estimates (e.g. regression coefficient) AND variation (e.g. standard deviation) or associated estimates of uncertainty (e.g. confidence intervals)
[ ]	[x]	For null hypothesis testing, the test statistic (e.g. *F*, *t*, *r*) with confidence intervals, effect sizes, degrees of freedom and *P* value noted<br>*Give P values as exact values whenever suitable.*
[x]	[ ]	For Bayesian analysis, information on the choice of priors and Markov chain Monte Carlo settings
[x]	[ ]	For hierarchical and complex designs, identification of the appropriate level for tests and full reporting of outcomes
[x]	[ ]	Estimates of effect sizes (e.g. Cohen's *d*, Pearson's *r*), indicating how they were calculated

Our web collection on statistics for biologists contains articles on many of the points above.

Software and code

Policy information about availability of computer code

Data collection	Flow cytometry acquisition: BD FACSDiva™ version 6.1.1, 8.0.2 and 1.4, Cytek SpectroFlo version 2.2.0<br>Sorting: BD FACSTM version 1.2.0.142
Data analysis	Flow cytometry analysis: FlowJo versions 9 and 10.7.1.<br><br>GraphPad Prism 8.4.3 and previous versions.

For manuscripts utilizing custom algorithms or software that are central to the research but not yet described in published literature, software must be made available to editors and reviewers. We strongly encourage code deposition in a community repository (e.g. GitHub). See the Nature Research guidelines for submitting code & software for further information.

Data

Policy information about availability of data

All manuscripts must include a data availability statement. This statement should provide the following information, where applicable:

- Accession codes, unique identifiers, or web links for publicly available datasets
- A list of figures that have associated raw data
- A description of any restrictions on data availability

The authors declare that the data supporting the findings of this study are available within the paper and its supplementary information files or are available from the authors upon reasonable requests. The Source data for Figures 1-5 and Supplementary Figures 1-5 are provided with the paper.

Field-specific reporting

Please select the one below that is the best fit for your research. If you are not sure, read the appropriate sections before making your selection.

[x] Life sciences [ ] Behavioural & social sciences [ ] Ecological, evolutionary & environmental sciences

For a reference copy of the document with all sections, see nature.com/documents/nr-reporting-summary-flat.pdf

Life sciences study design

All studies must disclose on these points even when the disclosure is negative.

Sample size	Group sizes were determined on the basis of preliminary experiments and were found sufficient to reveal biologically relevant differences in the samples of interest.
Data exclusions	Flow data samples were excluded from consideration if they failed quality checks such as including sufficient numbers of the cell population of interest and within the range expected for the sample being examined, if sample viability was below expected level for the material being examined, if sample acquisition was irregular due to equipment operation. Fewer that 1% of samples were excluded.
Replication	All experiments were repeated at least twice and and gave comparable results each time.
Randomization	Mice were allocated to experimental groups based on box numbers and were sex and age matched within experiments
Blinding	Investigators were not blinded to group allocation as data analysis involved in all cases objective measurement methods such as flow cytometry that are not affected by investigator bias.

Reporting for specific materials, systems and methods

We require information from authors about some types of materials, experimental systems and methods used in many studies. Here, indicate whether each material, system or method listed is relevant to your study. If you are not sure if a list item applies to your research, read the appropriate section before selecting a response.

Materials & experimental systems

n/a	Involved in the study	
[ ]	[x]	Antibodies
[x]	[ ]	Eukaryotic cell lines
[x]	[ ]	Palaeontology and archaeology
[ ]	[x]	Animals and other organisms
[x]	[ ]	Human research participants
[x]	[ ]	Clinical data
[x]	[ ]	Dual use research of concern

Methods

n/a	Involved in the study	
[x]	[ ]	ChIP-seq
[ ]	[x]	Flow cytometry
[x]	[ ]	MRI-based neuroimaging

Antibodies

Antibodies used

All monoclonal antibodies, Streptavidin reagents and Viability dyes used in this study, their clone numbers, catalog numbers (lot number/s), dilutions and validation information are provided below.

[BD Biosciences]

BCL6: K112-91, 561525 (6008775), 1:100, "Flow cytometric analysis of Bcl-6 expression in mouse B lymphocytes".
CD3: 145-2C11, 564379 (8339930), 1:200; 563565 (8249635), 1:200; 565992 (8249635) 1:200, "Two-color flow cytometric analysis of CD3e expression on mouse splenocytes".
CD4: RM4-5, 563151 (8179959), 1:200, "Two-color flow cytometric analysis of CD4 expression on mouse splenocytes".
CD4: GK1.5, 553729 (41619931), 1:1000, 563790 (6203974), 1:200, "Multicolor flow cytometric analysis of CD4 expression on mouse splenocytes".
CD8: 53-6.7, 560182 (3308752) 1:100, 563332 (7214673), 1:1000), 558106 (9346019), 1:400, "Two-color flow cytometric analysis of CD8a expression on mouse splenocytes".
CD11b: M1/70, 550993 (8232762), 1:500; 564443 (8206860, 8331604, 9346019), 1:200; 553311 (66680), 1:1000, "Flow Cytometric analysis of CD11b on mouse bone-marrow myeloid cells".
CD11c: HL3, 563735 (8043980), 1:100, "Flow cytometric analysis of CD11c expression on mouse dendritic cells".
CD40: 3/23, 553791 (4304865), 1:200, "Two color flow cytometric analysis of CD40 on mouse spleen cells."
CD44: IM7, 559250 (6113545), 1:400; 563058 (8144537) 1:200; 612799 (9028698), 1:400, "Flow cytometric analysis of CD44 expression on mouse bone-marrow cells".
CD45R (B220): RA3-6B2, 563793 (8269770), 1:200; 553093 (3354876), 1:100, "Flow cytometric analysis of CD45R expression on

mouse splenocytes".
CD86: GL1, 565479 (7193621, 9212704), 1:800; 564199 (7307761, 9262380), 1:200, "Flow cytometric analysis of CD86 expression on resting or activated mouse splenocytes".
CD103: M290, 557493 (7339822), 1:200; 561043 (2124837), 1:200, Flow cytometry (routinely tested) by BD.
CD185 (CXCR5): 2G8, 551960 (7068617, 8080503), 1:100, "Flow cytometric analysis of CD185 expression on mouse splenocytes".
CD273 (PDL2): TY25, 564245 (5232564), 1:200, "Flow cytometric analysis of CD273 expression on mouse splenocytes".
CD326: G8.8, 563134 (8072848, 9094621), 1:1600, "Flow cytometric analysis of BALB/c mouse thymocytes and splenic leukocytes".
Gata3: L50-823, 560068, (7216782), 1:100, "Comparison of GATA3 expression in human T and B cell lines".
IFNg: XMG1.2, 554411 (8099787, 9085768, 9197282), 1:200; 566097 (6043664), 1:100, "Flow cytometric analysis of stimulated (SEB) CD8+ and CD8- BALB/c spleen cells".
IL-4: 11B11, 554435 (9022663), 1:200, 554436 (75631), 1:100, "Flow cytometric analysis of BALB/c spleen cells stimulated with SEB for 72h and restimulated for 5h with anti-CD3 and anti-CD28 in the presence of monensin. Co-stained with anti-mouse CD4 FITC".
Ly6A/E (Sca1): D7, 562730 (8345830), 1:2000, "Flow cytometric analysis of Ly-6A/E expression on BALB/c mouse splenocytes (Concanavalin A-activated)".
Ly6C: AL-21, 563011 (7153555), 1:400, "Flow cytometric analysis of Ly6C expression on mouse splenic leukocytes."
Ly6G: 1A8, 563978 (8199527), 1:200, "Flow cytometric analysis of Ly6G expression on mouse bone marrow leukocytes."
MHCII: M5/114.15.2, 563413 (8228563), 1:400-1:800, "Two-color flow cytometric analysis of I-A/I-E MHC class II alloantigen expression on C57BL/6 mouse splenocytes".
NK1.1: PK136, 553165 (5191593), 1:400, "Flow cytometric two-color analysis of C57BL/6NHsd splenocytes with anti-mouse CD3 FITC".
RORgt: Q31-378, 562894 (8151861), 1:100, "Multicolor flow cytometric analysis of RORγt expression in mouse thymocytes".
Streptavidin: 554060 (8137939), 1:2000; 564176 (7096544), 1:500-1:1000; 562284 (4085873), 1:1200; 563260 (6195577), 1:200, Flow cytometry (routinely tested) by BD.
TCR: H57-597, 553169 (8004614, 8248656), 1:200, 562840 (9283446) 1:200, "Flow cytometric analysis of a   TCR expression in spleen and thymus (BALB/c splenocytes)".

[BioLegend]
CD3: 145-2C11, 100320 (B268542) 1:1600, "Flow cytometric analysis of C57BL/6 mouse splenocytes".
CD11c: N418, 117318 (B269973, B283053), 1:200-500, "Flow Cytometric analysis on mouse splenocytes".
CD19: 6D5, 115504 (B276685), 1:200, "Flow Cytometric analysis on mouse splenocytes".
CD64: X54-5/7.1, 139304 (B270364), 1:200, 139314 (B257960), 1:100, 139322 (B251526), 1:200, "Flow cytometric analysis on C57BL/6 bone marrow cells stained with CD11b".
CD169: 3D6.112, 142404 (B178247), 1:200, "Flow cytometric analysis of C57BL/6 mouse splenocytes".
CD197 (CCR7): 4B12, 120108 (B231613), 1:200, "Flow cytometric analysis of C57BL/6 mouse splenocytes".
CD206 (MMR): C068C2, 141712 (B266866), 1:400, "Flow Cytometric analysis on thioglycollate-elicited BALB/c peritoneal macrophages".
CD279 (PD1): 29F.1A12, 135218 (B176749), 1:200; 109110 (B245159), 1:400 "Flow cytometric analysis of Con-A stimulated C57BL/6 splenocytes."
CD301b (MGL2): URA-1, 146804 (B266066), 1:200, "Flow Cytometric analysis on C57BL/6 bone marrow-derived dendritic cells".
CD317: 927, 127019 (B261198), 1:200, "Flow Cytometric analysis on C57BL/6 splenocytes stained with CD45R/B220 PE".
CD326: G8.8, 118212 (B264359), 1:400, "Flow cytometric analysis of mouse thymic epithelial stromal cell line TE-71."
CXCL9: MIG-2F5.5, 515606 (B289446), 1:200, "Flow cytometric analysis of IFN-g stimulated peritoneal macrophages, intracellular stained with CD11b FITC".
IFNg: XMG1.2, 505846 (B249252), 1:100, "PMA + Ionomycin-stimulated C57BL/6 splenocytes were stained with CD3 APC, fixed, permeabilized, and then stained with IFN-γ (clone XMG1.2) PE/Dazzle™".
Ly6C: HK1.4, 128014 (B214150), 1:1000-1600, 128018 (B247616), 1:1000, 128030 (B200177), 1:200, "Flow cytometric analysis of mouse bone marrow cells".
Ly6G: 1A8, 127604 (B270990), 1:400, "Flow cytometric analysis of C57BL/6 bone marrow cells".
MHCII: M5/114.15.2, 107622 (B264454), 1:100; 107620 (B252427), 1:400, "Flow cytometric analysis of C57BL/6 mouse splenocytes".
TCRβ: H57-597, 109206 (B241593), 1:200, "Flow cytometric analysis of C57BL/6 mouse splenocytes".
T-bet: 4B10, 644810 (B240425, B240426), 1:100, "Human peripheral blood lymphocytes were surface stained with CD3 APC and stained with T-bet (clone 4B10) PE or mouse IgG1, κ PE isotype control".
XCR1: ZET, 148216 (B249983, B279176), 1:200, "Flow cytometric analysis of CD11c+ cells from collagenase-digested C57BL/6 mouse spleen".
Zombie NIRTM Fixable Viability Kit: 423106, 1:1000, "Flow cytometric analysis of one day old C57BL/6 mouse splenocytes".

[ThermoFisher Scientific]
CD4: GK1.5, 25-0041-82 (4280800), 1:200; 47-0041-82 (4313102), 1:800, "Staining of C57BL/6 splenocytes with Anti-Mouse CD19 and 0.06 μg of Anti-Mouse CD4".
CD19: eBio1D3, 13-0193-82 (1924620), 1:200, "Staining of C57BL/6 splenocytes with Anti-Human/Mouse CD45R (B220) APC and 0.125 μg of Anti-Mouse CD19 Biotin followed by Streptavidin PE".
CD44: IM7, 56-0441-82 (E08986-1634), 1:200, "Flow cytometric analysis of BALB/c splenocytes (lymphocyte gate)".
CD185 (CXCR5): SPRCL5, 46-7185-82 (4307893), 1:50, "Staining of BALB/c splenocytes with Anti-Mouse CD19 and Anti-Mouse CD185".
DAPI (4',6-Diamidino-2-Phenylindole, Dihydrochloride): D1306, 0.1 μg/mL, Flow cytometric analysis of splenocytes.
IL-17A: eBio17B7, 25-7177-82 (4300417, 2093758), 1:200, "Flow cytometric analysis of 10-day Th17-polarized mouse splenocytes and stimulated with Cell Stimulation Cocktail".
Live Dead   Fixable Aqua: L34965, 1:200 – 1:1000, "Flow cytometric analysis of Jurkat cells labelled with the LIVE/DEAD   Fixable Aqua Dead Cell Stain Kit."
Live Dead   Fixable Blue: L34962, 1:1000, "Flow cytometric analysis of Jurkat cells heat treated at 60 degrees celsius."

[Grown and labeled in-house]
CD4: GK1.5, AF647, 1:500, Flow cytometric analysis of CD4 expression on C57BL/6 splenocytes.
CD8: 2.43, AF647, 1:1000, FITC, 1:1000, Flow cytometric analysis of CD8 expression on C57BL/6 splenocytes.
MHCII: 3JP, AF647, 1:1000, Flow cytometric analysis of MHCII expression on C57BL/6 splenocytes.
Hybridoma cell lines were originally obtained from the Basel Institute for Immunology, Basel, CH (GK1.5 and 2.43) and NIH, Bethesda, USA (3JP), and verified for specificity by flow cytometric analysis of spleen cells from the appropriate mouse strains.

[BioXCell]
InVivoPlus anti-IFNγ: XMG1.2, BP0055 (609216A2B), 500μg/dose by ip injection, validated by previous studies listed at https://bxcell.com/product/invivoplus-anti-m-ifngamma/
InVivoPlus anti-IL-12p40: C17.8, BP0051 (717619A1), 500μg/dose by ip injection, validated by previous studies listed at https://bxcell.com/product/invivoplus-anti-m-il-12-il-23/
InVivo anti-IL-18: YIGIF74-1G7, BE0237 (715119F1), 300μg/dose by ip injection, validated by previous studies listed at https://bxcell.com/product/anti-m-il-18/
InVivoPlus IgG1 isotype, anti-horseradish peroxidase: HRPN, BP0088 (592816M2), 500μg/dose by ip injection.
InVivoPlus IgG2a isotype: 2A3, BE0089 (716719M1), 500μg/dose by ip injection.

Validation

For commercially available antibodies, validation was performed by the manufacturer and their statements are cited in the table above.
For antibodies generated in-house, validation and titration studies were performed by flow cytometric analysis of C57BL/6 splenocytes as stated above.

Animals and other organisms

Policy information about studies involving animals; ARRIVE guidelines recommended for reporting animal research

Laboratory animals

Mice were bred and housed under specific pathogen-free conditions at the Malaghan Institute of Medical Research (ambient temperature 21±3°C, humidity 50±10%, light/dark cycle 12/12h) or the NIAID, NIH (ambient temperature 22± 3°C, humidity 50±20%, light/dark cycle 14/10h). All animals were between 6-14 week old and age- and sex-matched within experiment. C57BL/6J (000664), CCR2-KO (004999), Il12b-YFP (006412), Itgax-cre (008068) and Irf4fl (009380) mice were originally obtained from the Jackson Laboratory (Bar Harbor, ME); BATF3-KO and Langerin-DTR mice were from Dr. Kenneth Murphy (Washington University, St Louis, MO) and Dr. Bernard Malissen (Centre d'Immunologie de Marseille-Luminy, Marseille, France), respectively. For some experiments, CCR2-KO mice (8456) and C57BL/6Tac control animals were obtained from the NIAID contract facility at Taconic Farms and Taconic Biosciences (Rensselaer, NY) , respectively. Itgax-cre+/- Irf4fl/fl or fl/- (IRF4ΔCD11c) and Itgax-cre-/- Irf4fl/fl or fl/- (IRF4WT) mice were obtained from Dr. William Agace (Lund University, Sweden) and bred at the University of Manchester Biological Services Facility. Bone marrow from IRF4ΔCD11c, IRF4WT or C57BL/6J mice was used to reconstitute lethally irradiated (two doses of 500cGy) C57BL/6 recipients. After reconstitution, chimeras were rested for 10+ weeks before immunization. All experimental procedures were performed in accordance with institutional guidelines.

Wild animals

This study did not involve wild animals

Field-collected samples

This study did not involve samples collected from the field.

Ethics oversight

This study was approved by the Animal Ethics Committee of Victoria University of Wellington, NZ, and by the NIAID Animal Care and Use Committee of NIH, Bethesda, USA, as detailed in Material and Methods.

Note that full information on the approval of the study protocol must also be provided in the manuscript.

Flow Cytometry

Plots

Confirm that:

- [x] The axis labels state the marker and fluorochrome used (e.g. CD4-FITC).
- [x] The axis scales are clearly visible. Include numbers along axes only for bottom left plot of group (a 'group' is an analysis of identical markers).
- [x] All plots are contour plots with outliers or pseudocolor plots.
- [x] A numerical value for number of cells or percentage (with statistics) is provided.

Methodology

Sample preparation

For assessment of T cell intracellular cytokines, LN single cell suspensions were prepared by gently pressing LN through a 70μm cell strainer, washed with IMDM, and cultured in foetal calf serum-supplemented IMDM (Invitrogen) in the presence of 50ng/mL PMA (Sigma-Aldrich), 1μg/mL ionomycin (Merck Millipore) and 1μL/mL GolgiStop™ (BD Pharmingen) for 5 hours at 37°C.

DC were prepared from auricular LN by digesting with 100µg/mL Liberase TL and 100µg/mL DNase I (both from Roche, Germany) for 25 minutes at 37°C before passing through a 70µm cell strainer. For assessment of cytokine production by DC, monocytes and NK cells, single cell suspensions were incubated in foetal calf serum-supplemented IMDM in the presence of GolgiStop™ (BD Pharmingen) and Brefeldin A (Sigma Aldrich) for 6 hours.

Instrument

All samples were collected on a LSRFortessa SORP™, LSRII SORP™, FACSymphony SORP™ flow cytometer (all from Becton Dickinson) using BD FACSDiva™ software, or an Aurora spectral cytometer (Cytek Biosciences).

Software

Flow cytometry acquisition: BD FACSDiva™ (BF); Spectroflo (Cytek)

Flow cytometry analysis was with FlowJo software (version 10, Treestar Inc).

Cell population abundance

Relevant populations were greater than 95% of sorted populations as determined by reanalysis of a sample of sorted cells.

Gating strategy

The gating strategies used in this study are reported in Supplemental data. Specifically, gating for CD4+ T cells is shown in Supp Figure 1a, gating for Ag+/Ag- dendritic cell subsets is shown in Sup Figure 1c and gating used for sorting Ag+/Ag- dendritic cell subsets is shown in Sup Figure 2.

All flow cytometry data presented in this paper passed quality control in FlowJo v10. Cells were pre-gated on singlets, using sequential SSC-A v FSC-W and SSC-A v SSC-W gates, and live cells, by gating out DAPI+ or Live/Dead fixable dye+ cells. Debris was excluded using an FSC-A v SSC-A gate.

CD4+ T cells were defined as CD3+ and/or TCRβ+, CD4+, CD8- (Sup Figure 1a). Total CD4+ T cells were assessed for expression of CD44, IFNγ, IL-4, IL-17A, Tbet, Gata-3 or RORγt. T follicular helper cells were defined as BCL6+ or CXCR5+ and PD1+ (Sup Figure 1b).

Dendritic cells were defined as B220- and/or CD19-, Ly6C-, CD11c mid-high and MHCII+. Monocytes were defined as B220- CD11b+ Ly6Chi CD64+. In some experiments, TCRβ+ and Ly6G+ cells were also excluded prior to DC/monocyte gating (Sup Figure 1c). Migratory DC were defined as CD11c mid-high and MHCIIhigh. MigDC subsets were identified as follows: LC (CD326+ XCR1-), DC1 (XCR1+), CD11b-high DC2 (CD326- XCR1- CD11b+), CD11b-low DC2 (CD326- XCR1- CD11blow) (Sup Fig 1d). Resident DC were defined as CD11chigh and MHCII mid. Resident DC subsets were identified as follows: DC1 (XCR1+), DC2 (XCR1- CD11b+).

Fluorescence minus one (FMO) controls assisted in defining positive and negative populations. PBS-injected mice served as staining controls for identifying Ag+ cells (Sup Figure 1c).

☒ Tick this box to confirm that a figure exemplifying the gating strategy is provided in the Supplementary Information.
